# Characterizing vascular function in mouse models of Alzheimer’s disease, atherosclerosis, and mixed Alzheimer’s and atherosclerosis

**DOI:** 10.1117/1.NPh.12.S1.S14610

**Published:** 2025-05-21

**Authors:** Beth Eyre, Kira Shaw, Dave Drew, Alexandra Rayson, Osman Shabir, Llywelyn Lee, Sheila Francis, Jason Berwick, Clare Howarth

**Affiliations:** a University of Sheffield, Department of Psychology, Sheffield Neurovascular Group, Sheffield, United Kingdom; b University of Sheffield, Neuroscience Institute, Sheffield, United Kingdom; c University of Sheffield, Healthy Lifespan Institute, Sheffield, United Kingdom; d Massachusetts General Hospital, Harvard Medical School, Department of Neurology, Boston, Massachusetts, United States; e University of Sheffield, School of Medicine and Population Health, Sheffield, United Kingdom

**Keywords:** Alzheimer’s, atherosclerosis, mixed disease, optical imaging spectroscopy, hemodynamic, vasculature

## Abstract

**Significance:**

Alzheimer’s disease does not occur in isolation, and there are many comorbidities associated with the disease, especially diseases of the vasculature. Atherosclerosis is a known risk factor for the subsequent development of Alzheimer’s disease; therefore, understanding how both diseases interact will provide a greater understanding of co-morbid disease progression and aid the development of potential new treatments.

**Aim:**

We characterize hemodynamic responses and cognitive performance in APP/PS1 Alzheimer’s mice, atherosclerosis mice, and a mixed disease group (APP/PS1 and atherosclerosis) between the ages of 9 and 12 months.

**Approach:**

Whisker-evoked hemodynamic responses and recognition memory were assessed in awake mice, immunohistochemistry to assess amyloid pathology, and histology to characterize atherosclerotic plaque load.

**Results:**

We observed hemodynamic deficits in atherosclerosis mice (versus Alzheimer’s, mixed disease, or wild-type mice), with reduced short-duration stimulus-evoked hemodynamic responses occurring when there was no concurrent locomotion during the stimulation period. Mixed Alzheimer’s and atherosclerosis models did not show differences in amyloid beta coverage in the cortex or hippocampus or atherosclerotic plaque burden in the aortic arch vs relevant Alzheimer’s or atherosclerosis controls. Consistent with the subtle vascular deficits and no pathology differences, we also observed no difference in performance on the object recognition task across groups.

**Conclusions:**

These results emphasize the importance of experimental design for characterizing vascular function across disease groups, as locomotion and stimulus duration impacted the ability to detect differences between groups. Although atherosclerosis did reduce hemodynamic responses, these were recovered in the presence of co-occurring Alzheimer’s disease, which may provide targets for future studies to explore the potentially contrasting vasodilatory mechanisms these diseases impact.

## 1 Introduction

Globally, there are over 55 million people living with dementia, with this set to increase to over 139 million by 2030.^1^ Alzheimer’s disease (AD) is the most common cause of dementia, with hallmark features including the deposition of amyloid beta (Aβ) in extracellular plaques^2^ and abnormal folding and aggregation of tau to intraneuronal fibrils.^3^^,^^4^ Accumulating evidence suggests that neurovascular dysfunction may be important in the pathogenesis of AD, with vascular changes being observed as one of the first pathological events in the disease.^5^ The two-hit vascular hypothesis^6^ suggests that vascular risk factors (e.g., diabetes, hypertension, cardiovascular disease, and/or cerebrovascular damage) damage the blood–brain barrier (BBB)^7^^,^^8^ and reduce cerebral blood flow,^9^^–^^11^ promoting the accumulation of Alzheimer’s amyloid-β toxin and hypoxia. The BBB, the brain’s largest exchange surface, serves as the primary clearance pathway for neurotoxic molecules such as amyloid-β.^12^ Brain capillary endothelial cells form a tight monolayer connected by adherens junction proteins, limiting the entry of harmful plasma-derived proteins, pathogens, red blood cells, and leukocytes into the brain.^12^ Disruption of the BBB allows neurotoxic products such as fibrin, thrombin, hemoglobin, and plasmin to enter the tissue, causing neuronal damage via direct neuronal toxicity, oxidative stress, or detachment of neurons from their supporting extracellular matrix.^13^^,^^14^ Disruption of the neurovascular control systems at the capillary level, including the ability to direct blood flow to active brain areas, would also be expected to increase hypoxia,^15^ which further upregulates the amyloid precursor protein.^16^^,^^17^ These Aβ-independent and Aβ-dependent pathways impair synaptic and neuronal function, ultimately leading to the cognitive impairment observed in AD.

Alzheimer’s is not a disease that occurs in isolation. As age is the greatest risk factor of the disease, with the vast majority of cases being sporadic and occurring in individuals aged 65 years and above,^18^ many individuals also possess comorbidities.^19^ Individuals who possess a greater number of comorbidities have poorer outcomes.^20^ AD and diseases of the vasculature share common risk factors, such as diabetes, hypertension, and hypercholesterinaemia,^21^^–^^24^ meaning research that assesses how vascular comorbidities impact neurovascular function is warranted. It is important to assess how comorbid disease may affect neurovascular function, especially within preclinical research as this could potentially explain why many animal studies assessing drug treatments for Alzheimer’s disease are unable to translate to humans,^25^^–^^27^ for instance if they focus on singular mechanisms, which are differentially modulated across disease types.

Atherosclerosis is the second biggest killer in the UK^28^ and can take decades to manifest clinically with patients being largely asymptomatic until their mid-50s.^29^ Atherosclerosis is an inflammatory disease caused by an excess of lipids within the blood, which are internalized by endothelial cells resulting in the progressive thickening and hardening of major artery walls.^30^ Although the deposition of lipids into the arterial walls is not sufficient to cause atherogenesis, research indicates that the subsequent immune response involving invading monocytes and leukocytes may initiate the syndrome.^31^ Atherosclerosis can result in reduced perfusion over time due to the occlusion of arteries, and many studies have observed associations between atherosclerosis and AD.^32^^–^^34^

Atherosclerosis can be modeled preclinically in a number of ways,^35^ although mice are highly resistant to atherosclerosis and exhibit vascular lesions following a high cholesterol diet, which differs from the human condition in the histologic nature and location, likely due to a chronic inflammatory state rather than genetic predisposition.^36^ Some preclinical methods of inducing atherosclerosis use genetic knockout mouse models such as the low-density lipid receptor (LDLR) −/−^37^ or Apolipoprotein E (APOE) −/−.^38^ Genetic mutations that cause a loss of function to either the LDLR, ApoE, or ApoB of an LDL molecule result in the reduced clearance of LDL from the plasma, which increases circulating plasma levels of cholesterol.^39^ Neurovascular deficits have previously been reported in the LDLR−/− model of atherosclerosis, where Lu et al.,^40^ reported weaker evoked-hemodynamic responses to a whisker stimulation, as well as hypoxic pockets in cortical tissue and microvascular changes in capillaries in atherosclerotic mice at 12 months of age (versus atherosclerotic mice at 3 months where no lesions were yet present in the aorta, carotid arteries, or cerebral arteries). In a separate study, this group further observed that in the cortex of 12-month-old atherosclerotic mice, tissue oxygenation, red blood cell velocity, and flux were decreased, and capillary diameters were smaller compared with younger counterparts.^41^ Furthermore, the ApoE−/− mice have been shown to develop BBB leakage, which worsens over time and may contribute to deficits in neurovascular coupling.^38^^,^^42^ However, breeding genetically modified mice can be time-consuming and expensive, especially when wanting to model atherosclerosis concurrently with other diseases. Recently, other ways of inducing atherosclerosis have been employed, via the use of viral vectors.^43^^–^^45^ Injecting a gain-of-function mutation of PCSK9 via a viral vector in addition to a Western diet can increase cholesterol levels^43^^,^^44^ and over time result in atherosclerotic lesions. The proprotein convertase subtilisin/kexin type 9 (PCSK9) gene on chromosome 1 (1p32.3) has been shown to homeostatically regulate cholesterol levels in the plasma by causing the internalization and degradation of the LDL receptor and related APOE receptor (ApoER2).^46^^–^^48^

Although animal models cannot fully replicate the complexity of human disease, particularly due to mice’s 2-year lifespan and differing lipid profiles (with cholesterol carried primarily on high-density rather than low-density lipoproteins),^36^ they remain valuable for preclinical modeling of comorbid pathophysiological events. Mice can develop atherosclerosis and/or Alzheimer’s pathology within a short time frame, offering insights into synergistic disease mechanisms. Previous work by Hohsfield and colleagues showed hypercholesterolemia (induced via a high cholesterol diet) impaired cognitive function, promoted glial cell activation, and altered vascular integrity but did not impact amyloid deposition and Alzheimer-like pathology in triple-transgenic (Psen1, APPSwe, tauB301L) mice up to 14 months old.^49^ Although it is worth noting that triple-transgenic mice on the high cholesterol diet did not survive beyond 14 months, meaning they may not have yet gained enough cumulative amyloid/tau deposition to manifest as Alzheimer-like pathology. By contrast, Grames et al.^44^ induced hypercholesterolemia through PCSK9 gene transfer in APP/PS1 Alzheimer’s mice and observed an increase in plaque burden in the hippocampus by 7 months. In recent research from our lab, Shabir et al. induced atherosclerosis in mice via intravenous injection of rAAV8-mPCSK9-D377Y to investigate whether atherosclerosis alone or in the presence of amyloid overexpression (J20-AD) can impact neurovascular function in a lightly anesthetized preparation.^45^ They observed that the presence of atherosclerosis resulted in a reduction in the size of the peak hemodynamic response to a 2s whisker stimulation. However, they observed no effect of AD alone or mixed Alzheimer’s and atherosclerosis on evoked-hemodynamic responses, which they speculated could be due to the presence of amyloid plaques invoking compensatory angiogenesis in cerebral microvessels to enhance perfusion in response to increased Aβ and neuroinflammation.

In the present study, we combined comorbid Alzheimer’s and atherosclerosis using the viral injection of a gain-of-function mutation of PCSK9 (with a Western diet) in the APP/PS1 mouse strain^50^^,^^51^ to assess how AD alone, atherosclerosis alone, and mixed AD and atherosclerosis impact recognition memory, amyloid pathology, and sensory-induced vascular function in the awake mouse.^52^ Although our experimental preparation to measure *in vivo* hemodynamic responses was not confounded by the impact of anesthesia on basal perfusion and vasodilation capacity,^53^ it is important to note that locomotion impacts hemodynamic activity and was accounted for in our analysis.^54^

## 2 Methods

### 2.1 Animals

Male mice aged between 9 and 12 months from the following groups were used: APP/PS1 [B6.C3-Tg(APPswe, PSEN1dE9)85Dbo/Mmjax #34829] Alzheimer’s model,^55^ referred to as AD throughout the manuscript; wild-type littermates referred to as WT, an atherosclerosis model [WT-littermates injected with rAAV8-mPCSK9-D377Y ($6\times 10^{12}\;\text{virus molecules}/\mathrm{mL}$) (Vector Core, Chapel Hill, NC) at 11 weeks of age (i/v or i/p + a Western diet at 12 weeks (21% fat, 0.15% cholesterol, 0.03% cholate, 0.296% sodium; #829100, Special Diet Services UK)] referred to as ATH; and a mixed disease group [APP/PS1 mice injected with rAAV8-mPCSK9-D377Y ($6\times 10^{12}\;\text{virus molecules}/\mathrm{mL}$) (Vector Core, Chapel Hill, NC) at 11 weeks of age (i/v or i/p + a western diet at 12 weeks)] referred to as MIX. Western diet began at 12 weeks and continued until the end of the study. The diet was tolerated well with no adverse effects observed through standardized laboratory pain measures administered in rodent experimental work (e.g., facial grimace scoring, piloerection, hunched posture, spontaneous activity in homecage), although it is worth noting that the diet resulted in some mice getting slightly oily fur. Consumption of the Western diet also had a significant impact on weight, measured on the second awake 2D-OIS imaging time point (mice aged between 9 and 11 months; one-way ANOVA main effect of group F(3,26)=8.76, p=0.0004), with Tukey *post hoc* tests indicating the ATH and MIX mice were significantly heavier than the AD mice. Prior to surgery, mice were housed in litters where possible; however, some were singly housed for welfare reasons or if there were no available littermates. A 12-h dark/light cycle (lights on 06:00–18:00) was implemented. Food and water were accessible *ad libitum* with the Western diet limited to 5 g per day per mouse. Experiments were completed during the light cycle, between the hours of 8 am and 4 pm.

At 9 months of age, APP/PS1 mice have an abundance of amyloid plaques, within the cortex and hippocampus,^56^ in addition to also showing cognitive deficits.^57^^–^^59^ Previous studies have given the AAV8-PCSK9 injection from as early as 30 days old up to 9 weeks old^45^^,^^60^^–^^62^ and initial studies investigating the use of AAV8-PCSK9 with a Western diet revealed that atherosclerosis can be induced in 12 weeks.^43^ We injected mice at 11 weeks old and started the diet at 12 weeks old to ensure that mice would develop atherosclerosis by the start of behavioral experiments at 9 months of age.^45^

For the two groups with induced atherosclerosis, we only collected data from male mice due to differential expression of mPCSK9 in the livers of males and females,^63^ the estrous cycle stage and menopause status correlating with circulating PCSK9 levels,^64^^,^^65^ and females showing overall higher PCSK9 concentrations versus males.^64^^,^^66^

Procedures used in the study were approved by the UK Home Office and in agreement with the guidelines and scientific regulations of the Animals (Scientific Procedures) Act 1986. Further approval was also granted by the University of Sheffield licensing committee and ethical review board. The following study is reported in accordance with the ARRIVE guidelines E10 checklist. Once mice with the appropriate date of birth were identified, the selection was randomized. The experimenter was blinded from the disease group where possible during both the collection and analysis. A priori sample sizes were not conducted.

### 2.2 Novel Object Recognition Task

**Fig. 1:**
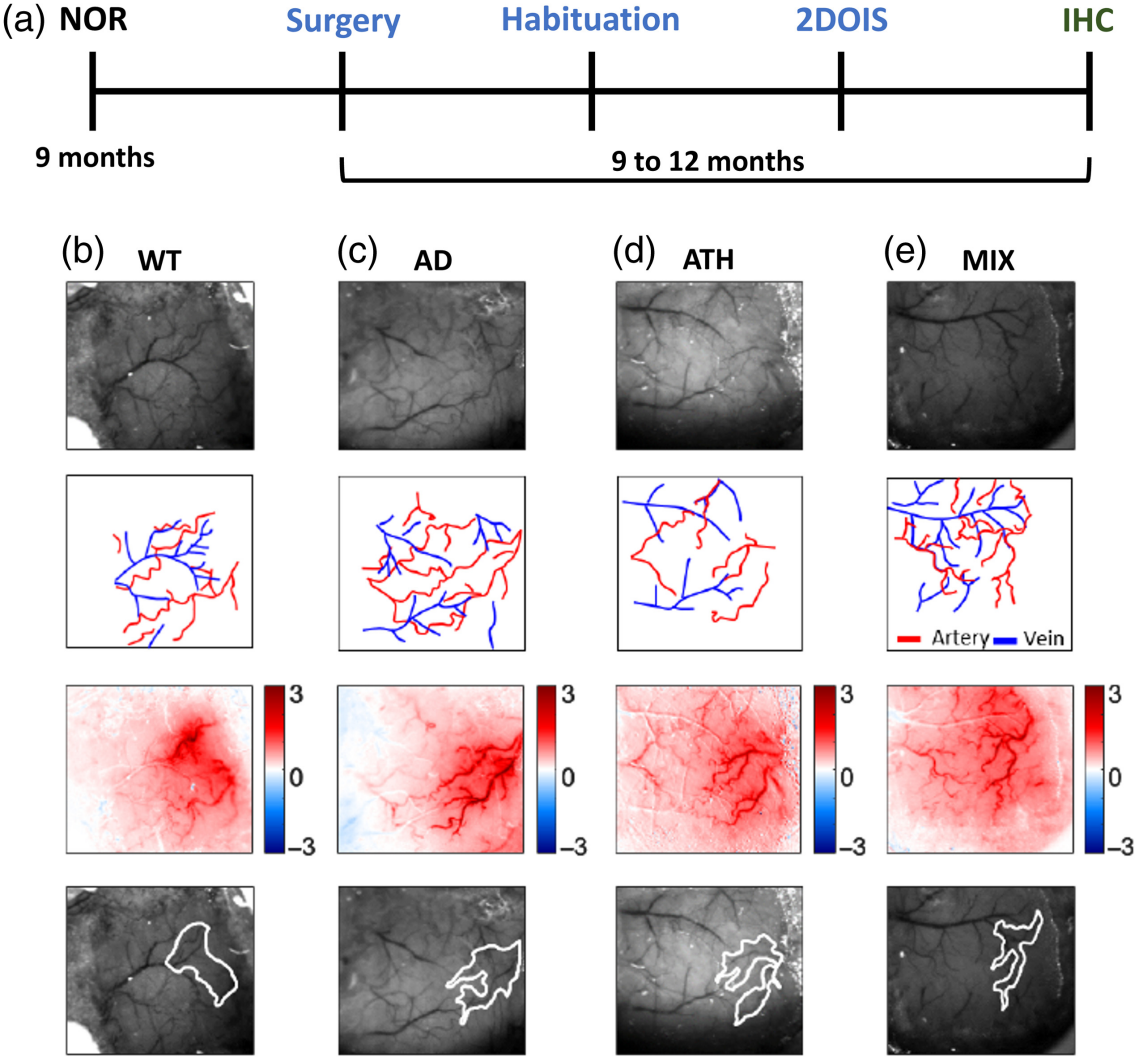
Experimental timeline and representative 2D-OIS spatial images. (a) Experimental timeline for all mice included in these experiments. Subjects first underwent cognitive testing on the novel object recognition (NOR) task at 9 months, before a surgery to insert a headplate and thinned window over the whisker barrel cortex was conducted. After a recovery period (minimum 1 week), habituation to the imaging apparatus was completed (5 days) and regional hemodynamic responses were recorded in awake mice using two-dimensional optical imaging spectroscopy (2DOIS). Mice were sacrificed using transcardial perfusion, and immunohistochemical (IHC) staining was conducted to assess amyloid coverage in AD and MIX groups. Histology was also conducted on the ATH and MIX groups to assess atherosclerotic plaque burden in the aortic arch. Representative animal from each disease group (b) WT, (c) AD, (d) ATH, and (e) MIX revealing the thinned window region (row one), vessel maps revealing arteries and veins (row two), a spatial map of HbT changes to a 2-s whisker stimulation (row three) with red colors indicating an increase in HbT in response to a 2-s whisker stimulation, and the region of interest overlying the active whisker barrel region (row four, white ROI).

At 9 months of age, non-spatial recognition memory was assessed using the novel object recognition (NOR) test [Figs. 1(a) and 5]. A 2-day protocol was used based upon the protocol developed by Lueptow.^67^ On day 1, the habituation phase took place. Mice were placed into a square open field arena (40  cm×40  cm×40  cm) for 10 min, and behavior was recorded using a camera placed above the arena. Day 2 consisted of the training and testing phases. In the training phase, mice were placed into the open field arena with two identical objects (either two glass beakers or two Duplo tower blocks—these were counterbalanced across mice). Objects were placed diagonally from each other; the positioning of objects was also counterbalanced across mice. After 10 min, mice were removed from the arena. After a 1-h retention period,^57^^,^^68^^–^^70^ mice were placed back into the arena. However, one of the familiar objects had been replaced with a novel object. Mice were handled for ∼7 days prior to cognitive testing to reduce experimental stress. The arena and objects were cleaned with 70% ethanol between mice. At least 30 min before cognitive testing, mice were moved to the experimental room to habituate them to the new room and to reduce stress. Time spent with each of the objects was measured using EthoVision software (EthoVision XT 15, Noldus). Mice were deemed to be exploring an object if their nose was <2  cm away from the object. However, climbing on top of objects was not considered exploration and was not included as exploration of objects. Distance traveled and velocity during training and testing were also recorded. Exploration of individual objects allowed for the calculation of the preference index. The preference index was calculated by dividing the time spent with the novel object by the total exploration of both objects multiplied by 100 (during the testing phase). This produced a percentage score of preference for the novel object, with a score of 50% suggesting no preference for the novel object, a score >50% indicating a preference for the novel object, and a score <50% indicating a preference for the familiar object. To be included in the analysis mice had to explore both familiar objects (in the training phase) for a minimum of 20 s each.

### 2.3 Surgery

Surgery to implant a head plate and thinned cranial window was conducted between 9 and 12 months of age [Fig. 1(a)]. Briefly, anesthesia was induced using ketamine (50  mg/kg) and medetomidine (0.65  mg/kg) (subcutaneously injected, s/c). The surgical plane of anesthesia was maintained with the addition of isoflurane (0.5% to 0.8% in 100% oxygen). Carprofen (10  mg/kg, s/c) was administered prior to removal of hair from the head. Animals were placed in a stereotaxic frame (Kopf Instruments) and ophthalmic gel was administered (Viscotears, Novartis). Body temperature was constantly monitored and sustained using a rectal thermometer and a homeothermic blanket (Harvard Apparatus). Iodine and bupivacaine (50 to 100 mcL at 0.025%) were applied prior to exposing the skull. Suture lines were covered with cyanoacrylate glue, and a dental scraper scored the contralateral side of the skull. The bone overlying the right somatosensory cortex was thinned to translucency ($∼4\;{\mathrm{mm}}^{2}$) using a dental drill. Saline was administered throughout to cool the area and to assist with the visualization of the pial vasculature. Cyanoacrylate glue was applied to the thinned region. The metal head plate was attached using dental cement (Superbond C & B; Sun Medical). Atipamezole (2  mg/kg in 0.3 mL warm sterile saline s/c) was administered at the end of the procedure to reverse the effects of medetomidine. Following surgery, mice were placed in an incubator (29°C) and monitored. After surgery, mice were given at least one week to recover prior to habituation and awake imaging. Mice were closely observed for weight loss and signs of pain for 3 days after surgery and administered carprofen jelly (10  mg/kg) for at least 1 day.

### 2.4 Two-Dimensional Optical Imaging Spectroscopy (2D-OIS) in Awake Mice

After a minimum 1-week post-surgical recovery period mice underwent hemodynamic imaging using 2D-OIS at 9 to 12 months [Fig. 1(a)]. The awake imaging setup was previously described in Eyre et al.;^54^ however, a modified habituation and imaging procedure was used in the current study. Briefly, 1 week after surgery, mice were habituated to the awake imaging cylindrical treadmill set-up. Over 5 days, mice were familiarized with the experimental room, experimenter, and awake imaging apparatus. On day 1, mice were placed on the cylindrical treadmill for 10 min, the room light was kept on, and mice were not head-fixed. On day 2, mice were head-fixed and placed on the cylindrical treadmill, lights were turned off, and 2s whisker stimulations were conducted using a mechanical plastic T-bar. Mice were on the cylindrical treadmill for ∼20  min. Day 3 was the same as day 2, with a supplementary “spontaneous” experiment conducted, where hemodynamic measurements were collected but no whisker stimulation was applied. Mice were on the cylindrical treadmill for ∼30  min. Day 4 followed the same procedure as the preceding two days; however, an additional 16 s whisker stimulation experiment was conducted. Mice were on the cylindrical treadmill for ∼45  min to 1 h. Day 5 followed the exact same procedure as day 4. Mice received a reward of sunflower seeds following each experimental imaging day. Hemodynamic data were collected on all days where mice were head-fixed and used in the subsequent analysis [Figs. 1(b)–1(e)]. Mice were briefly anesthetized using isoflurane (3% to 4%) prior to being placed on the awake imaging apparatus. Mice were observed throughout using a thermal camera.

Locomotion behaviors were gathered using a cylindrical treadmill (Styrofoam ball, 20-cm diameter) with an optical motion sensor. In-house MATLAB scripts (MathWorks, 2024a) were used to analyze the locomotion data. The optical motion sensor logged treadmill movement during all experiments. Locomotion data files were composed of the locomotion data (a vector with zeros when the mouse was stationary and integers (arbitrary units) when the mouse was moving), a corresponding time vector (to capture frames per second), and the trigger points, which specified the explicit timing of whisker stimulations across trials for aligning across imaging modalities.

Changes in hemoglobin concentration to a 2 s or 16 s whisker stimulation were investigated in the surface vasculature using widefield imaging. Two-dimensional optical imaging (2D-OIS) uses four wavelengths of light to measure changes in oxygenated (HbO), deoxygenated (HbR), and total levels of hemoglobin (HbT). Cortical hemodynamics were investigated using four differential wavelengths of light (494±20  nm, 560±5  nm, 575±14  nm, and 595±5  nm). These wavelengths illuminated the thinned window region of the cortex [Figs. 1(b)–1(e), row 1], using a Lambda DG-four high-speed galvanometer (Sutter Instrument Company, United States). A Dalsa 1M60 CCD camera was implemented to capture remitted light at 184×184  pixels, at a 32-Hz frame rate, providing a resolution of ∼75  μm. 2D spatial maps of micromolar changes in HbO, HbR, and HbT were collected, which reveal changes in hemoglobin concentrations in the surface vasculature [Figs. 1(b)–1(e), row 3]. This is accomplished by implementing a path length scale algorithm (PLSA) to complete a spectral analysis. The PLSA uses the modified Beer–Lambert law, with a path-length correction factor, in addition to predicted absorption values of HbT, HbO, and HbR.^45^^,^^71^^,^^72^ Relative concentration estimates of the above were obtained from baseline values,^72^ where the hemoglobin concentration within the tissue was estimated as 100  μM and tissue saturation of oxygen within the whisker region estimated at 80% and artery region at 90%. These values are in line with published values calculated from ${\mathrm{pO}}_{2}$ measurements in awake^73^ and anesthetized mice.^74^

Regions of interest (ROIs) were generated from the previously described 2D spatial maps, using in-house MATLAB scripts.^45^^,^^71^^,^^72^ A whisker region was made using code that found the region of the cortex with the greatest change in HbT to a 2s whisker stimulation. Pixels were considered “active” if they had a value that was >1.5 standard deviation (STD) across the entire spatial map of the surface vasculature. The whisker ROI was therefore the region of the cortex in which there was the largest increase in HbT in response to a 2s whisker stimulation [Figs. 1(b)–1(e), row 4]. ROIs for an artery, vein, and parenchyma were also manually selected within the whisker region, with PCA used to identify the vessels with the greatest changes in HbT and HbR. ROIs were generated for each imaging session. Care was taken to select the same artery, vein, and parenchyma across imaging sessions for the same mice. Time series analyses in the current study were conducted on the active artery from within the whisker barrels (although are visualized for the overall whisker region or the vein or parenchyma within the whisker barrels in Figs. 1 and 2 in the Supplementary Material).

### 2.5 Hemodynamic Data Analysis

For 2-s and 16-s whisker stimulation experiments, hemodynamic responses (HbO, HbR, HbT) were cut into trials around the stimulation period. For 2 s experiments, these trials started 5 s before and 20 s after the onset of the stimulation, and for 16 s experiments, 10 s before and 60 s after the onset of the stimulation. All 2-s stimulation experiments consisted of 30 trials and 16 s experiments of 15 trials. Each trial was extracted individually alongside its corresponding locomotion trace. The locomotion traces were then classified as belonging to one of four categories: (1) no running during the stimulation, (2) running before but not during the stimulation, (3) running starts at the onset of stimulation, or (4) running before and during the stimulation. For this locomotion classification across trials, the running traces for the 4 s before and 4 s after stimulus onset/offset were assessed. For each individual trial, the area under the curve (AUC) of the hemodynamic and locomotion responses was assessed during and just beyond the stimulation period (5 to 10 s for 2s stimulation or 10 to 30 s for 16 s stimulation) using the “trapz” function in MATLAB, and the maximum peak by finding the largest value reached during this period. These time series metrics and locomotion group categorizations were then extracted in a table alongside the animal ID, disease group (WT, AD, ATH, or MIX), imaging session ID (days 1 to 4), and trial ID (1 to 30 for 2s, 1 to 15 for 16s) labels. Tables were then exported into R, where statistical analysis was conducted on individual trials using a linear mixed model. Only stimulation trials where there was no running (locomotion group 1) or where running started at the onset of the stimulation (locomotion group 3) were included in the main analysis due to the profound impact of locomotion on hemodynamic responses. In supplementary figures, expanding the investigation of the impact of locomotion on hemodynamic responses, all experimental trials were included and ranked in ascending order from least to most locomotion for the 5 s after stimulation onset (5 to 10 s) using the locomotion AUC metric previously described (Fig. 4 in the Supplementary Material).

### 2.6 Immunohistochemistry and Histology

At the end of the final experiment, mice were euthanized with pentobarbital (100 mg/kg, Euthatal, Merial Animal Health Ltd.). Cardiac perfusions were completed using saline (0.9%). A subset of mice were also perfused with formalin (10%). Brains were dissected, and the whole brain (or half) was either placed in formalin (10%) or snap-frozen in isopentane and stored in a −80°C freezer. Formalin-fixed brains were paraffin-embedded (FFPE) and were cut into coronal slices (5 to 7  μm) using a vibratome. Coronal sections were mounted onto slides for subsequent immunohistochemistry. An avidin-biotin complex (ABC) method was used to stain and quantify amyloid coverage. Briefly, slides were dewaxed with xylene and rehydrated using ethanol (100%, 100%, 95%, 70%) for 5 min each. Peroxidase activity was inhibited by placing in a bath of 3% $H_{2}O_{2}/12\;\mathrm{mL}$ methanol for 20 min. Following this, slides were placed in 70% formic acid for 10 min. Antigen retrieval was completed using a microwave oven in a buffer of trisodium citrate (PH6) for 10 min. After antigen retrieval, slides were placed in a bath of ${\mathrm{dH}}_{2}O$ for 10 min. Slides were washed in PBS (for 5  min×2). Slides were then incubated with 1.5% normal serum (goat) for 30 min at room temperature and then incubated with the primary antibody (Amyloid Beta Rabbit Monoclonal—1:500, Abcam, ab201060) overnight at 4°C. Slides were again washed in PBS (for 5  min×2), and the secondary antibody was applied for 30 min at room temperature. Slides were then washed with PBS (for 5  min×2). A horseradish peroxidase avidin-biotin complex (Vectastain Elite Kit, Vector Laboratories, UK) was then applied and left at room temperature for 30 min. A final wash in PBS (for 5  min×2) was completed and then 3,3-diaminobenzidine tetrahydrochloride (DAB) (Vector Laboratories, UK) was applied to help visualize the antibody (left for 3 min). Slides were rinsed with ${\mathrm{dH}}_{2}O$ for 3 to 5 min and counterstained with hematoxylin and differentiated with Scotts Tap Water. Finally, slides were dehydrated using baths of ethanol (70%, 90%, 100%, 100%) for 30 s each and placed in xylene for 2 to 3 min. Following dehydration, slices were mounted with coverslips using DPX. Stained slides were scanned using a slide scanner (3Dhistech Panoramic 250) at a 40× magnification.

A classifier model in Qupath was used to assess amyloid burden within the brain tissue.^75^ To create the classifier model, a high resolution (0.88  μm/pixel) random trees pixel classifier was trained on a subset of stained brain sections. To train the classifier model, a training image was created with cortex and hippocampus regions from eleven different sections. Using the wand tool, on each section within the larger training image, areas were classified as either “Aβ” (1149 annotations), “tissue” (415 annotations), or “ignore” (318 annotations). Once all sections within the training image had been classified with the above classifications, this information was used to train the pixel classifier. A high-resolution (0.88  μm/pixel) random trees pixel classifier was applied, after which a visual check was completed using the live prediction tool, which showed how well the pixel classification model was able to detect Aβ, tissue, or regions to ignore. When upon visual inspection, the model was able to distinguish well between the above classifications the model was saved and then applied to the remaining samples. For the cortex ROI, scanned images were viewed using the hematoxylin channel, and a rectangle ROI was drawn on an area of the cortex above the hippocampus. For the hippocampus ROI, the DAB channel remained on and an ROI was drawn around the entire hippocampus. The classifier was applied, and then, a manual inspection was conducted. Upon manual inspection, if areas of tissue outside the ROI were included, these were reclassified as “ignore.” In addition, if cerebral amyloid angiopathy (CAA) was classified as plaque, this was manually reclassified as “CAA” and any CAA not detected by the model was manually added. If poorly perfused vessels were classified as amyloid, these were reclassified as “tissue.” A subset of 14 sections was selected to analyze amyloid burden, three of these were excluded due to damaged tissue and/or the wrong region of the hippocampus and an additional two were removed as they were from female mice. Amyloid burden included both amyloid plaques and CAA, and this was calculated by the following formula:

$$\text{Amyloid burden}\;\%\;\text{area}=\;\frac{\text{Detected amyloid pixels}+\text{detected}\;\mathrm{CAA}\;\text{pixels}\;(\text{reclassified})+\text{added caa pixels}}{\text{Detected tissue pixels}+\text{detected amyloid pixels}+\text{detected}\;\mathrm{CAA}\;\text{pixels}\;(\text{reclassified})}\times 100$$

### 2.7 Identification and Quantification of Atherosclerosis

After cardiac perfusion, the heart and aorta were also dissected for atherosclerotic and mixed disease mice. The heart and aorta were blunt dissected and placed in formalin for a minimum of 24 h before being placed in PBS. They remained in PBS until they were stained and embedded into wax-filled Petri dishes. Visible fat was removed from the aorta under a dissecting microscope, and the aorta was cut from the heart at the aortic root. Dissecting scissors were used to cut the aorta down the middle to open the aortic arch. This allowed the aorta to be laid flat and pinned to wax in a Petri dish post-staining. Oil red O (Sigma, O0625-100G) was used to stain for natural triglycerides and lipids within the aortic arch. A solution of Oil red O (60% solution) was made using distilled water and isopropanol. Once the aorta had been cut it was stained. Briefly, the aorta was placed into distilled water (10 s), isopropanol (2 min), and then into the Oil Red O solution (6 min). It was then placed in isopropanol (2 min) and finally rinsed in distilled water. Following staining, Petri dishes were filled with wax. Once the wax had partially hardened 1-mm insect pins (Fine Science Tools, FST) were used to pin the edges of the aorta into the wax. A 12-MP camera placed 10 cm above the samples was used to take images of stained aortas. ImageJ was used to quantify the presence of atherosclerotic plaque burden in the aortic arch. The polygon tool was used to draw around the aortic arch (the ROI), and the area of this was measured. Images were converted to 8-bit grayscale images. A threshold was applied to identify positively stained Oil Red O areas. A threshold of 150 was applied to all samples. The percentage area of aortic arch plaque burden was calculated using the following equation:

$$\text{Atherosclerotic plaque burden}\;\%\;\text{Area}=\;\frac{\text{Positive pixels}\;(\text{black pixels})\;\text{within aortic arch}}{\text{Area aortic arch}}\times 100$$

Two aortas were excluded from the analysis due to significant damage to the aortic arch during the dissection.

### 2.8 Statistical Tests

Statistical tests were conducted in RStudio and GraphPad Prism (Version 10), and figures were created in GraphPad Prism and MATLAB (R2024a). The threshold for statistical significance was set at p≤0.05. All data are presented as mean ± SEM or individual data points unless otherwise stated. The number of animals and experimental trials are reported across all comparisons. Detailed statistical reports are included in SR1–SR6 in the Supplementary Material).

Multi-group comparisons (across disease group (WT, APP, ATH, MIX) only or disease group and trial number (1 to 30, 1 to 15) interactions) were conducted using linear mixed models, with animal ID inputted as the random factor to account for variations between groups being driven by a single outlier animal (lmer package RStudio). For behavioral data analysis (Fig. 5) where there was only one independent variable (disease group) and each animal only contributed one datapoint, a one-way ANOVA was conducted across all metrics (distance traveled, velocity, preference index) when data were normally distributed (assessed using a Shapiro-Wilks test), and a Kruskal-Wallis test was conducted when the data was not normally distributed (distance traveled during the testing phase). Where *post hoc* comparisons were conducted to explore significant effects, the Tukey method was used with correction for multiple comparisons. For correlational analysis exploring the relationship between the trial number or size of locomotion and hemodynamic responses (Fig. 3; Fig. S3 in the Supplementary Material), a Pearson’s R correlation was conducted to assess whether the variables were significantly linearly related and to compare the linear relationship between the disease groups a linear regression was conducted to compare the slope and intercepts.

## 3 Results

### 3.1 Atherosclerosis Mice Show a Reduced Hemodynamic Response to a Short 2 s Stimulation, But Not a Longer 16 s Stimulation

**Fig. 2:**
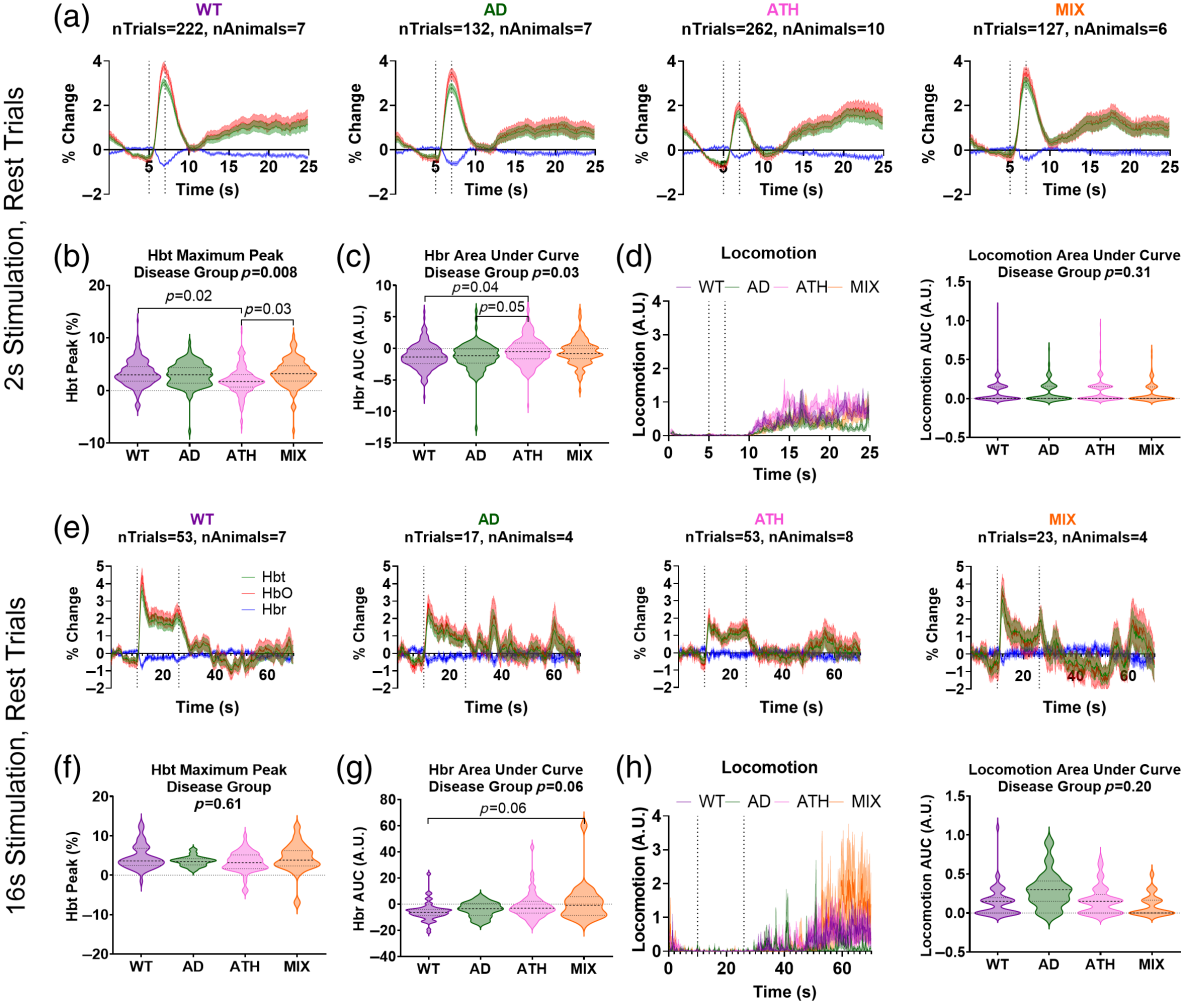
Comparing stimulation-induced responses without the confounds of locomotion. (a) Hemodynamic time series from the artery ROI within the whisker barrel region showing total (HbT, green), oxygenated (HbO, red), and deoxygenated (HbR, blue) hemoglobin in response to a 2-s mechanical whisker stimulation in trials with no concurrent locomotion occurring between the 4 s either side of the stimulus period (dotted lines) for wild-type (WT, purple), APP/PS1 (AD, green), atherosclerosis (ATH, pink), and mixed APP/PS1 × atherosclerosis (MIX, orange) mice. (b) The maximum peak of the HbT response during the stimulation period of these “rest trials” is compared across disease groups using a linear mixed model, with a significant overall effect of disease (p=0.008) driven by the atherosclerosis group (pink) showing smaller 2-s stimulus-induced responses than the wildtype (purple, p=0.02) or mixed (orange, p=0.03) mice. (c) There was a significant difference between disease groups on the area under the curve of the HbR response during the stimulation period of these “rest trials” (p=0.03), driven by the atherosclerosis group showing smaller HbR responses than the wild-types (p=0.04) and AD mice (p=0.05). (d) There were no differences in locomotion during these “rest trials,” assessed using the area under the curve of the locomotion response during the stimulation period (right). (e) Hemodynamic responses were also compared in response to a 16-s mechanical whisker stimulation in trials with no concurrent locomotion occurring between the 4 s on either side of the stimulation period (dotted lines) for the artery ROI. There was no significant difference in the size of the (f) HbT (maximum peak, p=0.61) or (g) HbR (AUC, p=0.06) responses between WT, AD, ATH, or MIX mice, although the HbR washout was smaller at trend level due to the mixed APP/PS1 × atherosclerosis mice showing smaller responses than wild-type. (h) There were also no differences in locomotion during rest trials for 16-s stimulation events. P-values are taken from linear mixed-effects models with disease group inputted as a fixed-effect factor, and animal ID as the random effect (lmer package RStudio), and pairwise comparisons (with correction for multiple comparisons) conducted using the Tukey method (emmeans package RStudio). Shaded error bars represent mean ± SEM. Horizontal lines on violin plots show median and interquartile ranges. The number of trials and animals included in each group are indicated on the time series graphs.

We compared short (2s) and long (16s) duration stimulation-induced hemodynamic responses from the artery within the whisker barrel region of somatosensory cortex across disease groups in rest trials where locomotion did not occur during the stimulation period (Fig. 2; SR1 in the Supplementary Material). In response to a 2-s whisker stimulation [Fig. 2(a)], there was a significant effect of disease on the maximum peak of the HbT trace [p=0.008; Fig. 2(b)] and the area under the curve of the HbR trace [p=0.03; Fig. 2(c)] as the atherosclerosis mice showed smaller hemodynamic responses than those in WT mice (WT versus ATH: HbT peak p=0.02; HbR AUC p=0.04). Of particular interest, the size of the HbT peak in atherosclerosis mice was significantly improved in the mixed model (ATH versus MIX p=0.03), replicating the findings of Shabir et al., who used the J20 model. These differences were not driven by differences in locomotion, as the confounds of locomotion were removed from this comparison by selecting only trials with no concurrent locomotion during the stimulation period [effect of disease on size of locomotion events: p=0.31; Fig. 2(d)]. By contrast, there was no significant effect of the disease group on the hemodynamic response induced by a 16-s whisker stimulation [Fig. 2(e)], as the size of the HbT maximum peak [Fig. 2(f)] and HbT area under the curve (linear mixed model: F=0.1567, p=0.92; data not shown) was comparable between groups. Although no overall significant difference was observed in the size of the 16-s stimulus-induced HbR response, there was a trend level effect of disease [p=0.06; Fig. 2(g)], as the mixed model showed a smaller HbR washout versus the wild-type animals. Again, locomotion did not differentially influence hemodynamic responses in the 16-s stimulation dataset as there was no difference between groups in the amount of locomotion during the stimulation period due to only rest trials being included [Fig. 2(h)]. Our results were replicated when we assessed 2 s and 16 s stimulation-evoked hemodynamic responses in our other regions of interest (the entire whisker barrel region, vein, and parenchyma) (Figs. 1 and 2 in the Supplementary Materials).

### 3.2 Trial Number Does Not Influence Stimulus-Evoked Hemodynamic Responses

**Fig. 3:**
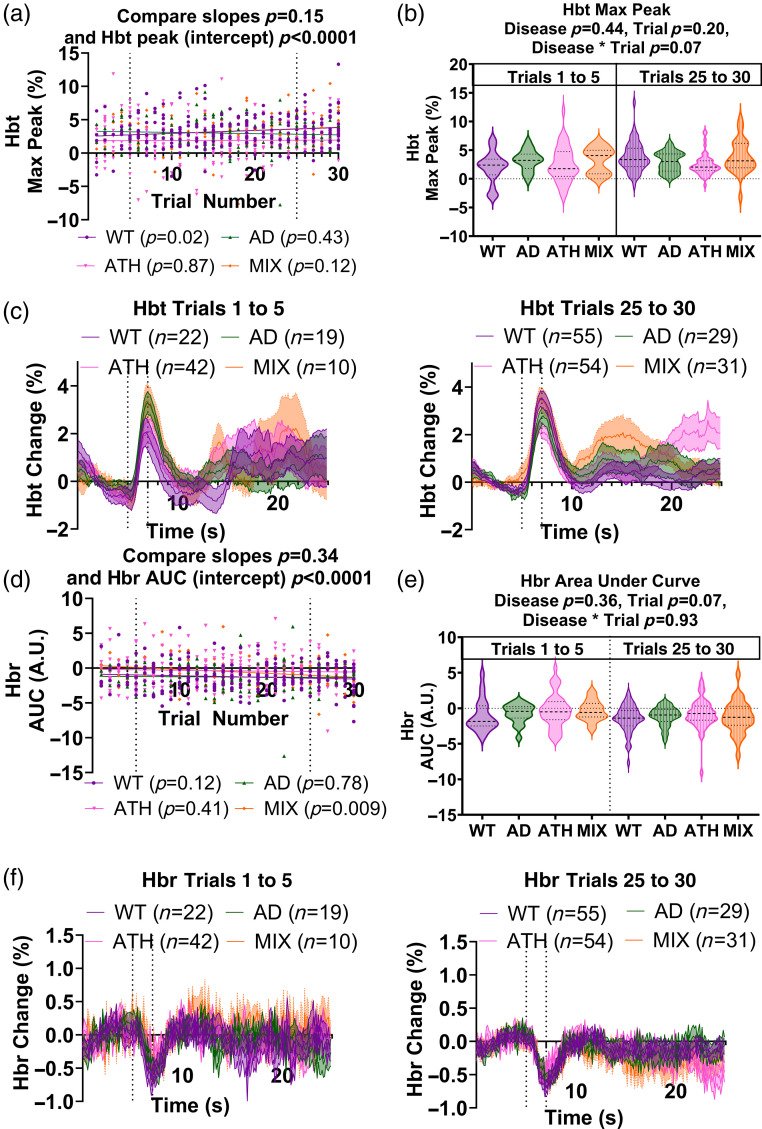
Differences across disease models in the size of 2-s stimulation responses during rest are not linked to the high number of trials. (a) There was no correlation between the trial number (1 to 30) and the size of the HbT response (max peak) for any of the disease groups (WT (p=0.02), purple; AD (p=0.43), green; ATH (p=0.87), pink; MIX (p=0.12), orange), although the WT mice showed a positive correlation whereby the size of the HbT peak increased with increasing trial number (purple, p=0.02). There was no significant impact of disease on the correlation between these two measures (compare slopes, p=0.15). However, there was an overall significant difference in the intercept (size of HbT peak) between disease groups (p<0.0001), with atherosclerosis mice consistently showing lower HbT peak values across all trials. (b) and (c) When HbT responses were separated as belonging to early (trials 1 to 5) or late (trials 25 to 30) trials and compared across disease and trial groups, there were no significant differences found. (d) There was also no correlation between the trial number (1 to 30) and size of the HbR response (AUC) for most of the disease groups [WT (p=0.12), purple; AD (p=0.78), green; ATH (p=0.41), pink], although the MIX mice did show larger HbR responses in later trials (p=0.009, orange). Overall, there was no significant impact of disease on the correlation between these two measures (compare slopes, p=0.34); however, there was an overall significant difference in the intercept (size of HbR AUC) between disease groups (p<0.0001), with WT mice consistently showing larger HbR AUC values across all trials. (e) and (f) When HbR responses were separated as belonging to early (trials 1 to 5) or late (trials 25 to 30) trials and compared across disease and trial groups, a significant effect of the trial was observed as HbR responses were larger in later trials. On scatterplots, individual values (dots) represent single trials, and p-values the result of a simple linear regression to test the correlation between variables within each disease group (legend) or the difference between groups on the slope and intercept (title). Shaded error bars represent mean ± SEM. Horizontal lines on violin plots show median and interquartile ranges. P-values on violin plots are taken from linear mixed-effects models with disease and trial group inputted as fixed-effect factors, animal ID as the random effect (lmer package RStudio), and pairwise comparisons (with correction for multiple comparisons) conducted using the Tukey method (emmeans package RStudio).

Given we observed differences between disease groups in the hemodynamic responses to a 2-s whisker stimulation but not to a 16-s whisker stimulation, we wondered whether the higher number of trials in the 2-s experimental paradigm (30 trials versus 15 trials for 16 s paradigm) could be driving our differences, for instance, the atherosclerosis group may be showing reduced hemodynamic activity only in later trials where the disease could be impacting the ability to sustain functional hyperemia (as seen in early aging by Balbi et al.^77^). As such, we investigated whether the trial number (with higher numbers indicating trials which occurred later in the experiment) impacted the size of hemodynamic responses to a 2-s stimulation (Fig. 3; SR2–SR3 in the Supplementary Material). We showed no differences in the relationship between the size of the HbT response (assessed using maximum peak) and the trial number across disease groups (p=0.15; linear regression analysis), although when HbT peak and trial number were correlated within each individual disease group the wild-type animals showed significantly increased HbT responses to later trials (p=0.02) where the other groups did not (individual results in figure legend). We also saw a significant difference in the intercept of the correlation line between disease groups (p<0.0001), as the atherosclerosis group intercepted the y-axis at a lower value, indicative of the smaller HbT response observed for this group [Fig. 3(a)]. To further explore the relationship between the size of the hemodynamic response and the trial number, we also visualized the average HbT traces for early (1 to 5) versus late (25 to 30) trials across disease groups and saw no effect of disease or trial number on the size of the HbT maximum peak, although there was a trend level interaction between disease group and trial number (p=0.07), driven by wild-type mice showing larger HbT responses in later trials [Figs. 2(b) and 2(c)]. The reason the overall effect of disease was lost in this comparison was likely due to the lower power to detect differences in this comparison due to the lower number of trials included (e.g., WT animals had 22 trials for the early group and 55 for the late group versus 222 trials in the overall comparison for Figs. 2, 3(a), and Fig. 3 in the Supplementary Material). When we conducted the same analysis on the HbR response using the area under the curve [Fig. 3(d) and 3(e)], we did see a relationship between the size of the HbR response and the trial number for the mixed (atherosclerosis × Alzheimer’s) group (p=0.009) but not the other groups (significance values for individual groups reported in figure legend), but no overall relationship between trial number and size of the HbR response across groups (p=0.34). Again there was an overall significant difference in the intercept of the HbR AUC between groups, as the atherosclerosis group showed smaller responses (p<0.0001) [Fig. 3(d)]. When the average HbR traces were visualized for early (1 to 5) and late (25 to 30) trials [Figs. 3(e) and 3(f)], there was no longer an overall impact of disease on the size of these responses, but there was a trend-level impact of trial number with the later trials showing larger decreases in HbR during the stimulation (p=0.07). The loss of the overall significant effect of disease in this comparison was again likely driven by the lower number of trials available reducing the statistical power for detecting differences between groups.

### 3.3 There are No Longer Differences Between Disease Groups in the Size of Stimulus-Evoked Hemodynamic Responses When Locomotion Co-occurs

**Fig. 4:**
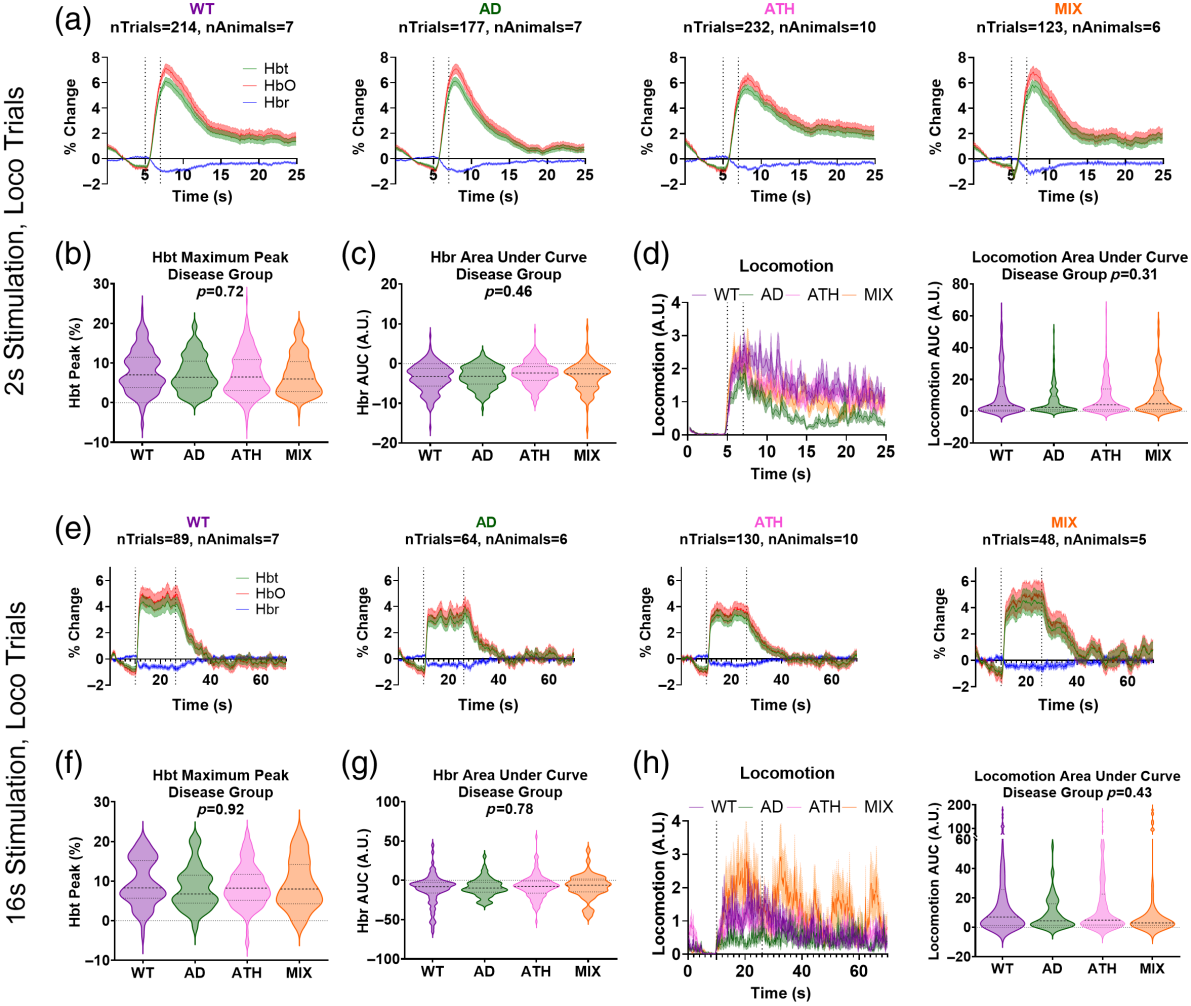
Comparing whisker stimulus-induced responses in trials that contain concurrent locomotion. (a) Hemodynamic time series from the artery region within the whisker barrels showing total (HbT, green), oxygenated (HbO, red), and deoxygenated (HbR, blue) hemoglobin in response to a 2-s mechanical whisker stimulation in trials with concurrent locomotion occurring at the onset of the stimulus period (dotted lines) for wild-type (WT, purple), APP/PS1 (AD, green), atherosclerosis (ATH, pink), and mixed APP/PS1 × atherosclerosis (MIX, orange) mice. There was no difference between disease groups in the size of the (b) maximum peak of the HbT response (p=0.72) or the (c) area under the curve of the HbR response (p=0.46) during the stimulation period of these “locomotion trials.” (d) There were no differences in locomotion across disease groups during these “locomotion trials,” assessed using the area under the curve of the locomotion response during the stimulation period (right, p=0.31). (e) Arterial hemodynamic responses were also compared for a 16-s mechanical whisker stimulation in trials with concurrent locomotion occurring at the onset of the stimulation period (dotted lines). There were no significant difference in the size of the (f) HbT (maximum peak, p=0.92) or (g) HbR (AUC, p=0.78) responses between WT, AD, ATH, or MIX mice. (h) There were also no differences in locomotion during “locomotion trials” for 16-s stimulation events (p=0.43). P-values are taken from linear mixed-effects models with disease group inputted as a fixed-effect factor, animal ID as the random effect (lmer package RStudio), and pairwise comparisons (with correction for multiple comparisons) conducted using the Tukey method (emmeans package RStudio). Shaded error bars represent mean ± SEM. Horizontal lines on violin plots show median and interquartile ranges. The number of trials and animals included in each group are indicated on the time series graphs.

As we have previously demonstrated that locomotion can impact hemodynamic responses profoundly,^54^ we also assessed stimulus-evoked hemodynamic activity in response to a 2- or 16-s stimulation in trials where locomotion co-occurred at the onset of the stimulus. Where we observed differences between groups to a 2-s stimulation presented in the absence of locomotion (Fig. 2), when locomotion was also present, there were no longer any differences between groups [Fig. 4(a); SR4 in the Supplementary Material], indicating that the vasculature of the atherosclerosis group does have the same capacity to dilate as the other groups but does not show as large responses to a sensory stimulus when locomotion is not present. Specifically, we observed no difference in the size of the maximum peak of the HbT response [Fig. 4(b)] or the area under the curve of the HbR response [Fig. 4(c)]. Because locomotion impacts hemodynamic responses^54^ (Fig. 3 in the Supplementary Material), we plotted the average locomotion traces for trials where locomotion started at the onset of stimulation for each disease group and confirmed that there was no difference in the size of locomotion events between groups [Fig. 4(d)]. We conducted the same analysis for the longer duration of 16-s whisker stimulation [Fig. 4(e)] and again saw no differences among groups in the size of the HbT maximum peak [Fig. 4(f)], HbR area under the curve [Fig. 4(g)], or locomotion events [Fig. 4(h)].

To confirm our method of locomotion analysis was not skewing our findings regarding hemodynamic responses across disease groups to a 2-s whisker stimulation, we also explored another method of locomotion classification where trials were ranked in ascending order from least to most locomotion occurring during the stimulation period- and the bottom and top 20% of locomotion trials were compared (similar to the analysis conducted by Eyre et al.;^54^ Fig. 4 in the Supplementary Material). Using this alternative method, we replicated our finding that differences were observed between disease groups only in the trials where locomotion was not confounding responses.

### 3.4 Subtle Neurovascular Deficits are Consistent with No Effect of Disease on Recognition Memory Assessed by the Novel Object Recognition Test

**Fig. 5:**
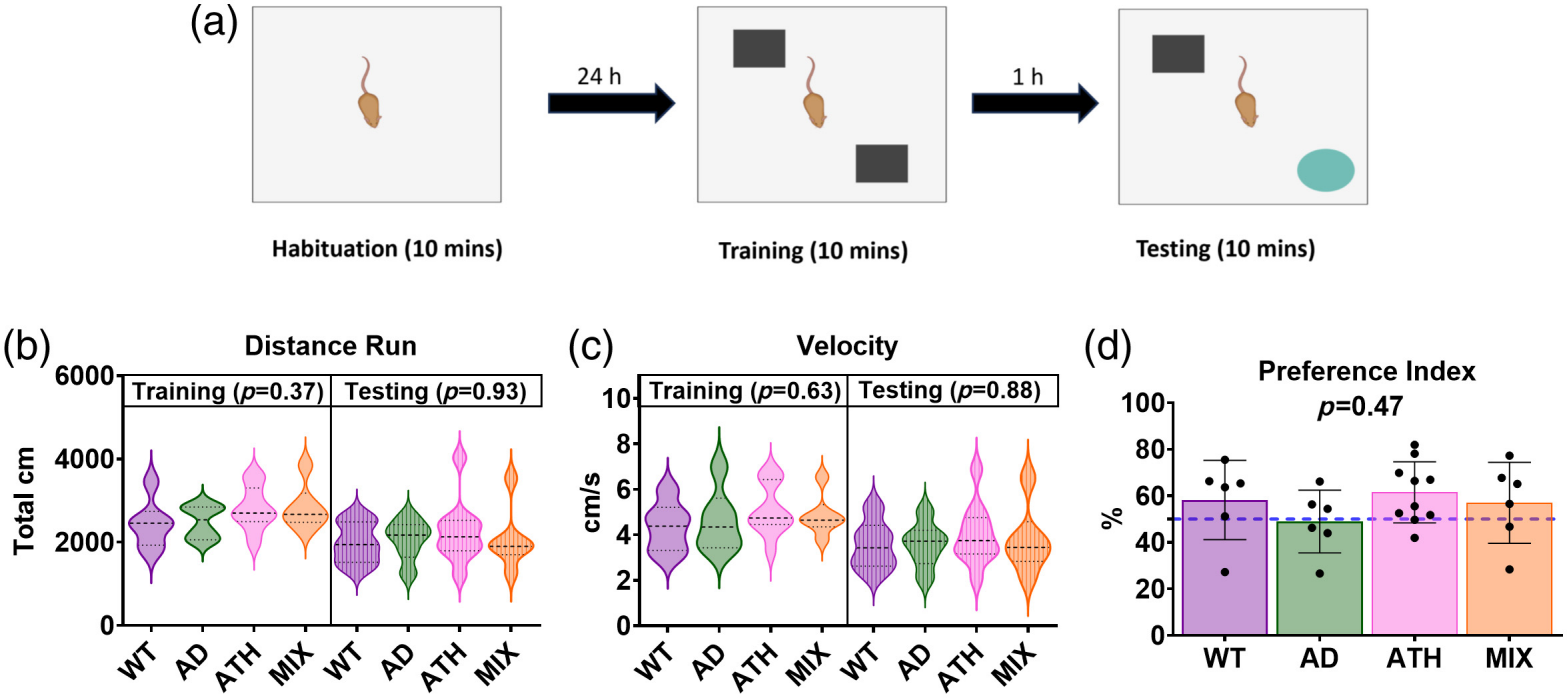
No differences in performance on a novel object recognition task between disease groups. (a) Mice were placed in a 40  cm×40  cm arena and allowed to freely explore for 10 min, which during the training phase (left) contained two identical objects and during the testing phase (right) included a novel object to replace either the left or right familiar object from the previous training session (Figure created in BioRender Ref. 76). Video recordings were taken of mice in the arena, and they had to explore each object for a minimum of 20 s during the training phase to be included in subsequent analysis. We compared the (b) distance run (cm) and (c) velocity (cm/s) of each mouse in the training and testing arena to indicate whether mice were engaging with the task similarly between disease groups. We showed no significant difference in the amount of time or speed of travel between groups. (d) To indicate the mouse’s preference for the novel versus familiar object in the testing arena, the preference index is calculated as [cumulative duration with novel object/total exploration time (cumulative duration with familiar + novel objects)] × 100. Values over 50% indicate the mouse has distinguished between familiar and novel objects and spent more time exploring the novel item. The wild-type, atherosclerosis, and mixed groups all had values >50% indicating a preference for the novel object, and a one-way ANOVA revealed no overall difference between groups on this task. Where groups showed a normal distribution, p-values are taken from one-way ANOVAs with group (WT, AD, ATH, MIX) as the independent variable and task performance metric (distance, velocity, preference) as the dependent variable. As the dataset for the distance run training phase was not normally distributed (assessed using a Shapiro-Wilks test), a Kruskal-Wallis comparison was run in place of the one-way ANOVA. Shaded error bars represent mean ± SEM. Horizontal lines on violin plots show median and interquartile ranges. Individual dots on bar charts represent single mice, the wild-type group included six mice, AD group six mice, atherosclerosis group 10 mice, and mixed group six mice across all figure panels (c)–(e).

We used the novel object recognition (NOR) test to assess recognition memory across groups. Mice were placed into an open field arena and could explore two of the same objects (training phase). After a 1-h delay, they were placed back into the same arena, with one of the previous objects replaced with a novel object (testing) [Fig. 5(a)]. Mice have an innate preference for novelty so therefore should want to spend more time with the novel object. Behavioral software was used to assess the time spent with each object [Fig. 5(b)], to assess the activity [Figs. 5(c) and 5(d)] and preference index [Fig. 5(e)] across groups. Activity was assessed using the distance run [Fig. 5(c)] and velocity of running [Fig. 5(d)] during the NOR training or test session, and we observed no difference between groups indicating that the mice engaged with the task to a similar degree. A higher preference index score indicates a greater preference for the novel object (with scores above 50% indicating performance on the task to distinguish between two objects is above chance). We found no significant differences in the preference index score across disease groups (WT – M: 58.2, SD: 17.1; AD – M: 48.9, SD: 13.5; ATH – M: 61.5, SD: 13.1; MIX – M: 56.9, SD: 17.4; one-way ANOVA F(3,24)=0.88, p=0.47). This suggests that there were no significant differences in the preference that mice had for the novel object—indicating that recognition memory was unchanged in AD, atherosclerosis, and mixed disease mice, as compared with WT mice, at 9 m of age, with a 1-h retention interval between training and testing. However, it is worth noting that a 48.9% preference index for the AD mice means that they did not perform above chance overall on the NOR task, which could indicate a subtle effect of disease on recognition memory, although our Tukey pairwise comparisons did not show these mice to be performing differently to the other groups (see SR5 in the Supplementary Material; AD-WT p=0.71, AD-ATH p=0.39, AD-MIX p=0.79).

### 3.5 Mixed Disease Does Not Enhance Atherosclerotic Plaque Burden or Amyloid Beta Load

**Fig. 6:**
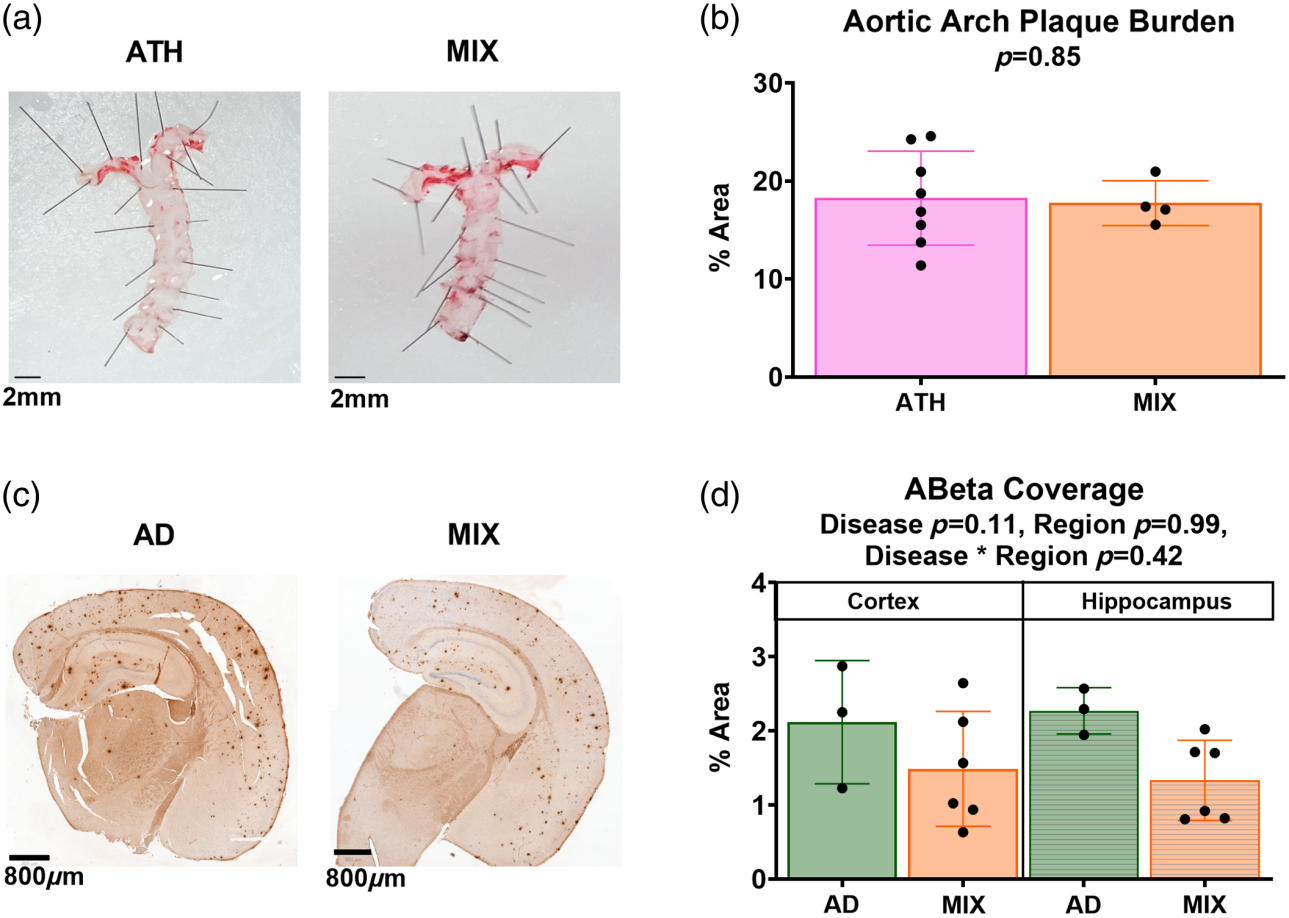
No differences in pathology across disease models. (a) Representative images of the aorta from an atherosclerotic mouse (left) and a mixed disease mouse (right) from which the percent plaque burden was calculated as the density of plaques found within the area of the aortic arch (top of the image where the aorta branches). The scale bar represents 2 mm. (b) The atherosclerosis (N=8) and mixed disease (N=4) mice showed no differences in plaque burden at the aortic arch (p=0.85). (c) Representative images of brain slices, which include CA1 hippocampus after labeling for amyloid beta in an Alzheimer’s mouse (left) and mixed disease mouse (right). The scale bar represents 800  μm. (d) Regions of interest were taken across the entirety of CA1 for the hippocampus, and for a rectangle overlying CA1 for cortex across all slices and the percentage of amyloid beta was calculated across the total area. There were no significant differences in amyloid coverage across our disease groups (AD N=3, MIX N=6, p=0.11) or brain regions (p=0.99).

As expected, we found atherosclerotic plaques within the aortic arch in atherosclerotic and mixed-disease mice [Fig. 6(a)]. We assessed whether the presence of amyloid beta within the brain may affect atherosclerosis in the systemic vasculature. We found no significant differences in aortic arch plaque burden between the atherosclerotic and mixed disease mice [Fig. 6(b), SR6 in the Supplementary Material]. Amyloid plaque burden was assessed using immunohistochemistry whereby the presence of amyloid was identified using an anti-amyloid antibody with DAB [Fig. 6(c)]. We assessed whether the presence of systemic atherosclerosis in addition to AD could impact amyloid coverage in the hippocampus or overlying cortex [Fig. 6(d); SR6 in the Supplementary Material]. We found no significant differences in amyloid coverage (calculated as % of the total area included in the analysis) when comparing AD mice to mixed disease mice (p=0.11) or between brain regions (p=0.99).

## 4 Discussion

In the current study, we investigated vascular and cognitive function in Alzheimer’s, atherosclerosis and mixed disease awake mouse models. We used 2D-OIS to investigate changes in cortical hemoglobin concentrations to external whisker stimulation with or without concurrent locomotion, immunohistochemistry to assess hippocampal and cortical amyloid, histology to assess aortic plaque load, and the NOR task as a test of non-spatial recognition memory.

### 4.1 Atherosclerosis Mice Showed Smaller 2-s Stimulus-Evoked Hemodynamic Responses During Rest

We observed that in trials in which concurrent locomotion occurred, there was no effect of disease on sensory-evoked hemodynamic responses. However, in support of our previous work, (showing the large effects locomotion can have on hemodynamic responses),^54^ we did find an effect of disease when we controlled for the confound of locomotion by selecting only trials where the animal was resting during the stimulation. When only rest trials were considered or when locomotion was ranked during the whisker stimulation, during the least locomotion trials (trials with no locomotion occurring throughout the stimulation period or bottom 20% of trials), we observed that whisker stimulation-evoked peak hemodynamic responses to a 2-s stimulation in the atherosclerosis group were significantly smaller than the wild-type and mixed disease mice. This supported previous work completed by our research group showing reduced stimulus-evoked hemodynamic responses in atherosclerosis mice only and therefore extended the findings that peripheral vascular changes resulting from atherosclerosis can subtly impact neurovascular function.^45^ It is possible that stimulus-evoked hemodynamic responses are reduced in chronic atherosclerosis due to the build-up of plaques inside extracranial and intracranial large arteries in the presence of elevated plasma low-density lipoprotein, which reduces or even blocks blood supply to the brain.^78^ The reduced stimulus-evoked hemodynamic response observed in atherosclerosis mice is also in alignment with studies from the Lesage group imaging 12- to 20-month LDLR−/− atherosclerosis mice, which showed weaker stimulus-evoked hemodynamic responses in the somatosensory cortex, with hypoxic micropockets in the cortical tissue recorded by two-photon phosphorescence lifetime microscopy,^40^ and reduced RBC flux, velocity, and hematocrit in individual capillaries.^40^^,^^41^ However, it is worth noting that these experiments lacked comparison to age-matched wild-type animals, leaving open the possibility that young atherosclerotic mice may have compensatory mechanisms temporarily enhancing hemodynamic responses or may not yet show age-related vascular impacts. In addition, alternative imaging methods were used, which were more sensitive to capillary bed changes, whereas our experiments recorded mainly from pial vessels leaving us unable to capture capillary-specific disease effects. In the second study by Li et al.,^41^ animals also underwent a more invasive preparation, including tracheotomy and cranial window placement immediately before anesthetized imaging under urethane. Anesthesia is known to affect hemodynamic responses,^53^ and older atherosclerotic mice may be more sensitive to such procedures due to more advanced neurodegeneration.^45^^,^^79^

Interestingly, although the atherosclerosis mice showed compromised hemodynamic responses to a 2-s stimulation in the current study, the hemodynamic responses were preserved to a longer 16-s stimulation (where HbT reached its maximum peak between 3.3 and 4.6 s after stimulus onset, which is outside of the short duration sensory stimulation period). Previous literature indicates that longer duration stimulation events involve alternative neurovascular signaling pathways, whereby when sensory activation is sustained (>3  s) delayed astrocyte calcium signals regulate the second phase of cerebral blood flow increases.^80^^–^^82^ However, it is also possible that in the awake imaging preparation, a higher number of trials is needed to provide a sufficient number of rest trials to uncover subtle differences, suggesting that our trial count may have been too low to detect group differences.

It is also interesting that combining AD with atherosclerosis in our hands seemingly “rescued” neurovascular responses, as we observed no reductions in hemodynamic responses in AD or MIX mice versus wild-type animals. It is possible that mild compensatory mechanisms in AD whereby angiogenesis is triggered in cerebral microvessels to compensate for mild hypoxia resulting from increased Aβ and neuroinflammation in the brain underlies the preserved responses in our AD and MIX models,^45^^,^^83^ although we have no measure of vascular density in these experiments and neovascularization in AD is eventually thought to be pathogenic and damaging to the brain due to enhanced endothelial Aβ secretion, which increases reactive oxygen species (ROS) levels.^83^ Future work would benefit from the inclusion of a measure of vascular density through the imaging of vessels labeled with a fluorescent dye using two-photon or confocal microscopy and an in-depth multi-omics investigation to explore potentially distinct vasodilatory mechanisms impacted across disease groups, which could potentially be targeted for benefit in disease.

Although it was surprising that we found no effects of AD or mixed AD and atherosclerotic disease on evoked-hemodynamic peak responses in this more “severe” APP/PS1 model of AD,^50^ previous investigations of hemodynamic responses in APP/PS1 mice, which have shown perturbations in stimulation-evoked hemodynamic responses had distinct methodological differences to this study. For instance, in previous work, which showed changes in hemodynamics in the APP/PS1 strain, Lu et al.^84^ showed reduced red blood cell velocity and hematocrit and reduced variations in velocity, flux, and hematocrit reflective of a reduction in capillary heterogeneity, as well as reduced stimulus-evoked oxygenated hemoglobin concentrations in the somatosensory cortex in APP/PS1 mice versus wild-type mice and APP/PS1 mice with access to an exercise wheel. However, these are pilot investigations with only four animals per group, and hemodynamic recordings were conducted in awake animals but locomotion was not accounted for during imaging, which we have previously shown to impact hemodynamic responses,^54^ and which could be occurring at different levels between groups. Work by van Veluw et al. also demonstrated a reduced arterial dilation to a visual stimulus in APP/PS1 mice at 9 to 12 months;^85^ however, the authors used a more invasive surgical procedure (craniotomy) and examined functional hyperemia in a different brain region (visual cortex). Other studies using two-photon microscopy in the APP/PS1 mouse model focused on capillary function and observed a larger number of “stalled” capillaries, which we were unable to capture with 2D-OIS.^86^ Given that we recorded net hemodynamic responses using 2D-optical imaging spectroscopy, it is possible our methodology was not sensitive to changes in individual vessels, and previous work by Nikolajsen et al.^87^ has shown that the structural integrity of the wider capillary network (capillary length, diffusion radius, pericyte number) is intact in aged (18 months) APP/PS1 mice, which is feeding the overall regional response. In the current study, we focused on evoked hemodynamic changes, which are summated in the surface pial vessels of the brain; therefore, it could be plausible that there are subtle effects of the disease at singular locations along the vascular tree.

### 4.2 Importance of Experimental Design

In the current study, we observed no effects of disease on evoked-hemodynamic responses when locomotion occurred concurrently with the stimulation. Previous research has evidenced that locomotion itself can increase hemodynamic responses within the brain.^52^^,^^54^^,^^88^^,^^89^ Our results show the importance of monitoring locomotion in awake mice, as there may be subtle vascular deficits across diseases, and recording locomotion to be able to dissect the effects of this behavior may allow the field to fully assess such subtle differences in vascular function across disease groups. Methods of data collection or analysis may need to be adapted to account for locomotion in future studies, with a potentially larger number of stimulation trials needing to be collected if trials with concurrent locomotion are to be removed (to retain sufficient statistical power to detect differences after data removal), or sophisticated analysis techniques applied to retain the higher number of trials and remove the confounds of locomotion on hemodynamic responses (e.g., using a hemodynamic response function to predict hemodynamic activity from spontaneous behaviors^90^ or applying a kernel analysis or method through which the locomotion-dependent hemodynamic response is subtracted from the hemodynamic response to whisker-stimulation alone).

Although including a large number of stimulus trials is important for accounting for the impact of locomotion (or rest) on hemodynamic responses, it may also allow for the assessment of fatigue, as aged or diseased mice may not sustain induced hemodynamic activity over time.^77^ Given we saw disease-related differences in the hemodynamic response for the 2-s whisker stimulation (30 trials) but not the 16-s whisker stimulation (15 trials), whereby the atherosclerosis mice showed smaller 2-s stimulus-induced responses, we wanted to ensure this was related to the shorter duration of the stimulus and not the higher number of trials collected. Previously Balbi et al.^77^ showed that older wild-type mice (8 to 12 months) were unable to maintain the size of the initial cerebral blood flow response compared to young mice (6 weeks) when a stimulation was presented up to 10 times. Here, we showed that there was no overall difference in the linear relationship between the size of the HbT response and the stimulus trial presentation number for the disease groups (AD, ATH, MIX), meaning the reduced 2-s stimulation-induced hemodynamic response in atherosclerotic mice was not due to an inability to sustain hemodynamic responses across repeated trials. Future work would benefit from randomizing the order of the stimulus protocol, as this could potentially control fatigue as well as stress-related effects.

It is important to ensure that animals are thoroughly habituated to the experimental apparatus and imaging paradigm,^91^^,^^92^ as stress has been shown to impact neurovascular responses.^93^ Furthermore, disease animals may have higher anxiety levels than wild-type animals,^94^ and stress can exacerbate disease pathology.^95^ In these experiments, mice were habituated to the experimental set-up over 5 days through increasing exposure^71^^,^^72^ and were monitored during imaging by the experimenter using a thermal camera to ensure stress behaviors (e.g., excessive head-pulling, piloerection, fecal boli production, vocalizations^96^) were not present. Future experiments would benefit from the recording of additional physiological parameters (e.g., whisking, pupil size) throughout the imaging session, as alongside locomotion these have also been shown to impact hemodynamic responses.^97^^–^^99^

### 4.3 Recognition Memory was Preserved Across All Groups, and Mixed Disease Did Not Enhance Pathology

We also found preserved recognition memory in all disease groups, as assessed using the NOR test, with a retention interval of 1 h (although AD mice did not perform above chance on this task, exploring the novel object for only 48.9% of the time spent in the arena). Finally, our immunohistochemistry experiments to assess amyloid plaque load found no difference in amyloid burden in the mixed disease group compared with the AD-only group, as has previously been reported,^44^^,^^45^ and no difference in plaque burden in the aortic arch of atherosclerosis or mixed disease mice. The similar amyloid plaque burden in the brain of AD and MIX mice was surprising given previous work from our group^45^ showed 3× more plaques in the MIX versus the AD mice; however, these differences could be due to the different AD mouse models used (in the previous study the J20 mouse, and here, APP/PS1, which has a more severe plaque load), as well as the different analysis methods. Rather than counting the number of plaques as per Shabir et al., we used a pixel classifier to identify amyloid.

We found no effect of disease on non-spatial recognition memory as assessed by the NOR test, which is in line with the subtle hemodynamic deficits we observed, although potentially surprising given a more “severe” model of AD was used. Although our behavioral results are not in line with some previous research in the APP/PS1 model, cognitive measures in mice often produce contradictory results, with some groups finding recognition memory deficits^57^^,^^68^ and other groups not.^69^^,^^100^ We also observed no differences in cognitive performance in our atherosclerosis or MIX mice (versus WT). This contrasts with previous studies in 7-month-old ApoE−/− mice,^101^ 12-month LDLR−/− mice fed a high cholesterol diet^102^^,^^103^ or 15 to 18 month APP mice fed an atherogenic diet,^104^ where anxiety behaviors were similar across all assessed groups, but the atherosclerosis mice showed significantly poorer performance on the Morris water maze task of spatial memory. It is possible contradictory findings arise from differences in the groups included (e.g., the sex of the mice, the age of the mice, drug treatments or not), the sample sizes used, and the metrics used to assess performance (e.g., time spent with an object, recognition index). In particular, cognitive deficits captured in the atherosclerosis mice from previous groups have assessed spatial memory using the Morris water maze, where we have focused on recognition memory. Furthermore, previous work has shown neurofibrillary tangles have a closer correlation with cognitive function than amyloid plaques,^105^ or that the effect of amyloid deposition on global cognitive function may be mediated by neurofibrillary tangles,^106^ a pathology that our mouse model did not produce. Limitations of the current behavioral analysis include the rigid exclusion criteria (two sessions were excluded, and in one session the camera did not record during the task) resulting in some of the groups having a smaller sample. In addition, the current study only investigated a retention interval of 1 h, whereas future work could use both shorter term (e.g., 3 min) and longer term (e.g., 24 h) retention intervals to provide a more extensive understanding of recognition memory in the above disease models. Applying a more extensive battery of cognitive tests (e.g., mood measured using nesting behavior, spatial learning, and memory through the Barnes maze and/or Y-maze) would provide a broader behavioral profile, which may be more sensitive to detect differences between groups.^107^

### 4.4 Limitations

To further elucidate the mechanisms underlying the observed differences in hemodynamic response, future work could use hypercapnia to further assess vasoreactivity, as previously performed in lightly anaesthetised^45^ and awake^108^ mice. Using the J20 AD model, Shabir et al.^45^ found no differences in hypercapnia-evoked hemodynamic response between disease groups (ATH, AD, MIX) and wild-type mice, indicative of preserved vasoreactivity. Furthermore, although we assessed aortic arch plaque burden in the atherosclerosis models, we did not examine carotid arteries for atherosclerotic plaques, which can induce cerebral hypoperfusion.^109^ Previous work assessing atherosclerotic plaque load in carotid arteries of wild-type animals injected with AAV-PCSK9 in addition to a high-fat diet and a partial ligation of one carotid artery suggests that the AAV-PCSK9 model of atherosclerosis alone does not result in atherosclerotic plaques within the carotid artery.^62^ However, these were C57 wild-type animals maintaining an alternative dietary regime for only 4 weeks. Thus, future work should investigate the effect of different diets for longer time periods. There is mixed evidence in the literature regarding whether basal CBF is altered in APP/PS1 mice with Lu et al. finding no difference in average RBC velocity and flux in brain capillaries of APP/PS1 mice versus WT,^40^ whereas Shen et al. found lower baseline cerebral perfusion in APP/PS1 AD mice versus WT as captured by ASL.^110^ As differences in basal CBF may influence relative neurovascular responses,^111^ altered basal CBF may, at least in part, underlie the differences that we have observed. Assessing cerebral perfusion and oxygenation across disease groups could have important implications for functional hyperemia responses, as reductions in oxygen saturation have been shown to diminish relative functional activation changes.^112^ These observations suggest caution when comparing hemodynamic responses across subjects whose baseline physiological parameters may differ. Future work should compare absolute basal CBF in these models to further our understanding of the cerebrovascular status of these models. It is also a limitation of the current study that no female mice were included, which was because female mice have been shown to respond differently to AAV8-PCSK9 injection, requiring three times the dose to induce hypercholesterolemia, which results in wider AAV8 tissue distribution and tropism.^63^ Following retro-orbital injection with AAV8-PCSK9, Vozenilek et al. observed PCSK9 mRNA in brain and heart tissue in female and male mice, suggesting possible central nervous system (CNS) effects (however, they could not detect PCSK9 protein within the heart or brain).^63^ Other studies also report that PCSK9 is differentially expressed between sexes^65^^,^^113^ and shows variability within females dependent on age^66^ and estrous cycle stage.^64^ Although the AAV-PCSK9 model of atherosclerosis has been shown to be an effective way of modeling atherosclerosis, the model can only be used to investigate fibrous plaques and not the effects of spontaneous plaque rupture,^114^ and it needs the addition of a western or high-fat diet to induce atherosclerotic lesions.^43^^,^^60^

### 4.5 Conclusion

This study provides an understanding of the impact that different neurodegenerative and vascular diseases can have on the vasculature in awake mice. We report deficits in the stimulus-evoked hemodynamic responses of atherosclerosis mice when the confound of locomotion is removed. Furthermore, our results emphasize the importance of experimental design when imaging awake mice, whereby stimulus duration should be considered and locomotion monitored.

## Supplementary Material

10.1117/1.NPh.12.S1. S14610.s01

## Data Availability

The data presented in this article are publicly available on ORDA at https://doi.org/10.15131/shef.data.27908352.v1.^115^ The individual hemodynamic and locomotion traces with corresponding labels (vessel group, disease group, imaging session, trial number) are available as MATLAB files, and the time series parameters used for the statistical calculations as a .csv file. The custom code used for data analysis is available as a GitHub repository (kirashaw1/CharacterizingVascFunction_ADATH), which has been published via Zenodo (https://doi.org/10.5281/zenodo.14238624). The outputs of all statistical analyses are reported in statistical tables SR1–SR6 in the Supplementary Material.
