# Supplementary material for: Characterizing vascular function in mouse models of Alzheimer’s disease, atherosclerosis, and mixed Alzheimer’s and atherosclerosis
Source: Neurophotonics. 2025 May 21;12(Suppl 1):S14610. doi: 10.1117/1.NPh.12.S1.S14610 (PMC12094910; doi:10.1117/1.NPh.12.S1.S14610)
Supplement: Supplementary file 1 [file NPh_012_S14610_SD001.pdf]

Appendix A: Supplemental Material

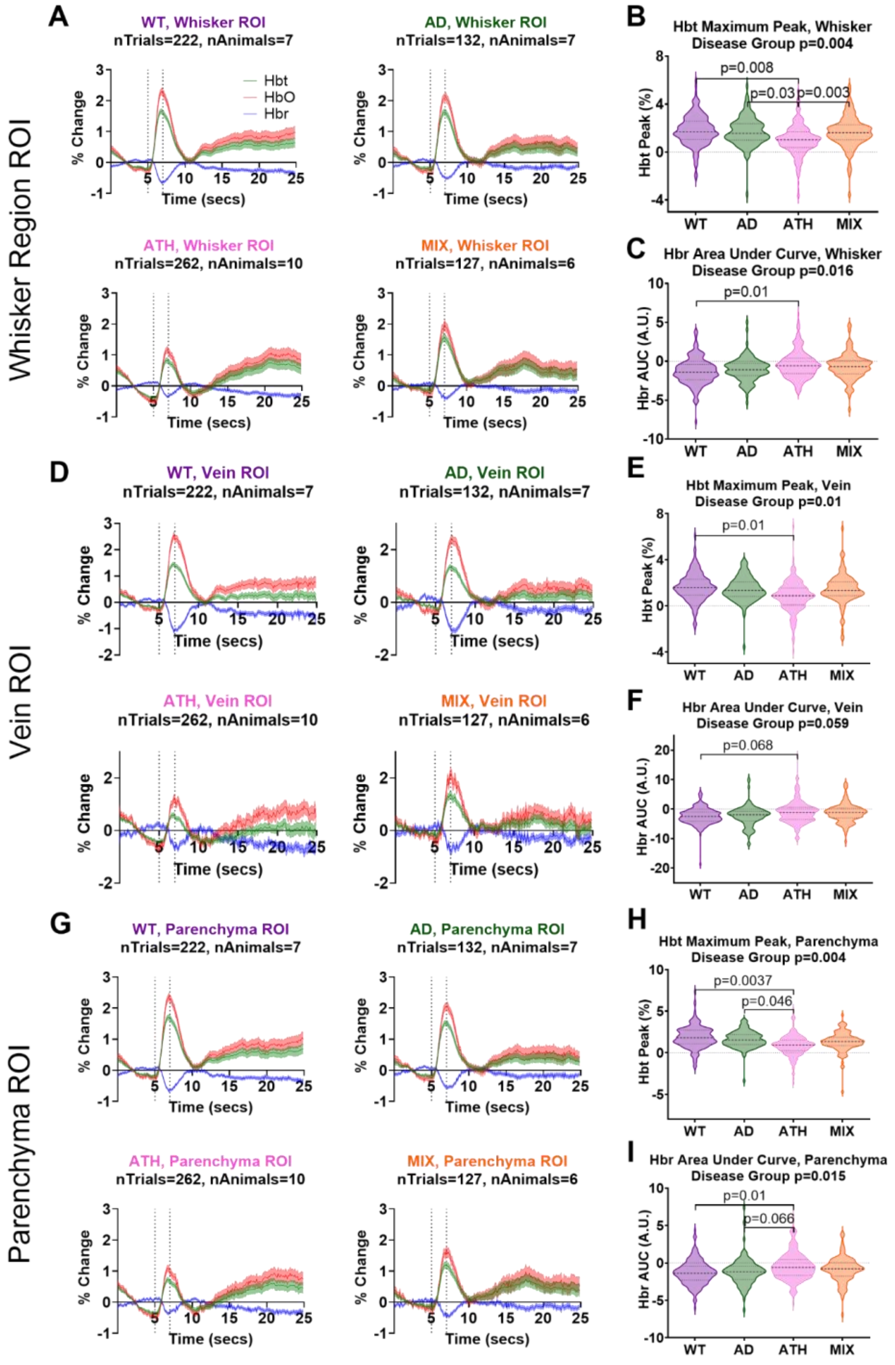

S Fig. 1 2s stimulus responses without the confounds of locomotion across the different vascular compartments.

Hemodynamic time series showing total (HbT, green), oxygenated (HbO, red), and deoxygenated (HbR, blue) hemoglobin in response to a 2s mechanical whisker stimulation in trials with no concurrent locomotion occurring between the 4 seconds either side of the stimulus period (dotted lines) for wild-type (WT, purple), APP/PS1 (AD, green), atherosclerosis (ATH, pink), and mixed APP/PS1 x atherosclerosis (MIX, orange) mice were also shown for the whisker (top, **A.**), vein (middle, **D.**) and parenchyma (bottom, **G.**) regions of interest. Findings were consistent with those observed in Figure 1, and showed a significant impact of disease group on the size of HbT and HbR responses which was driven by smaller responses in the atherosclerosis group (**B-C, E-F, H-I**).

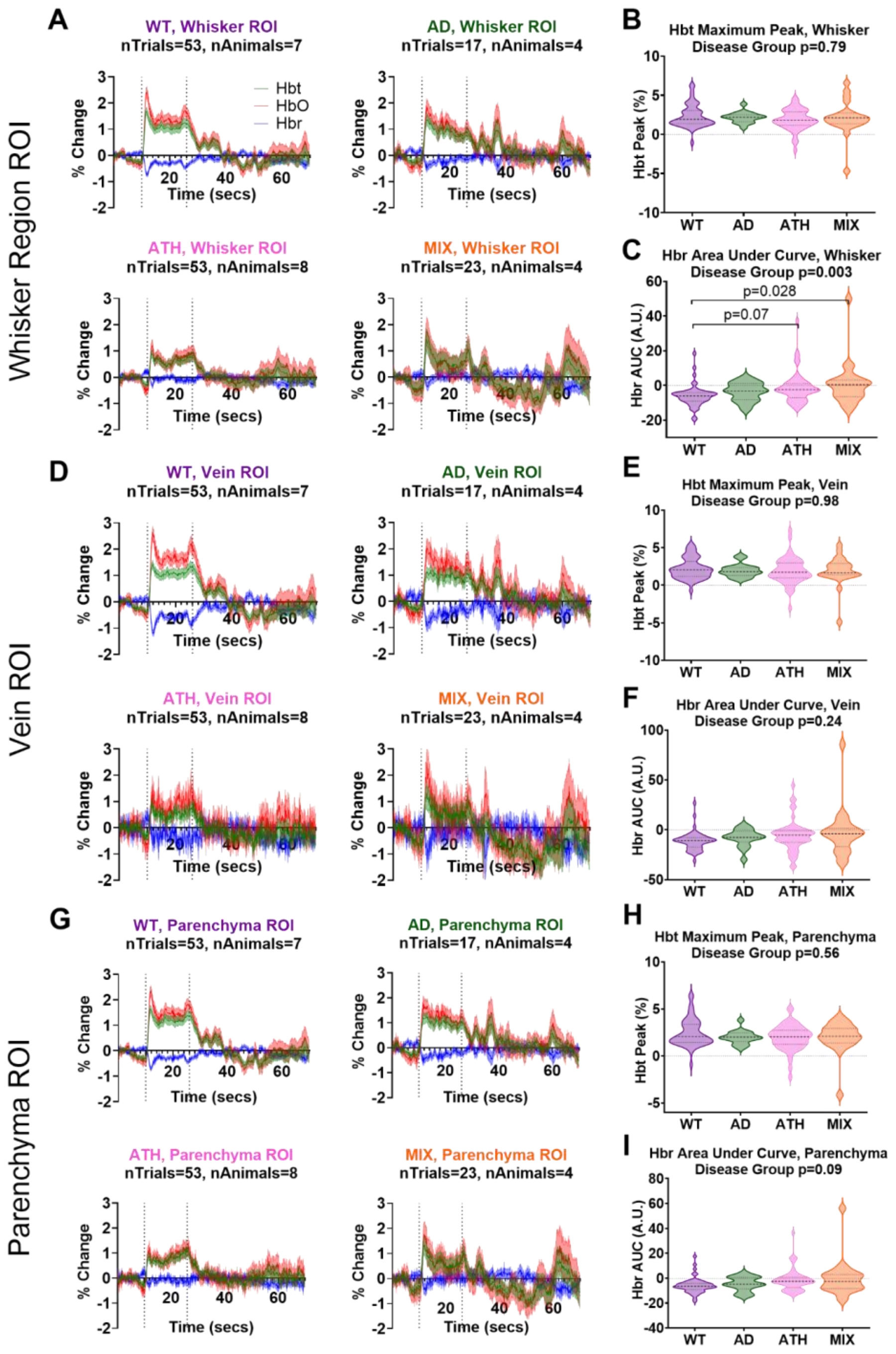

S Fig. 2 16s stimulus responses without the confounds of locomotion across the different vascular compartments.

Hemodynamic time series showing total (HbT, green), oxygenated (HbO, red), and deoxygenated (HbR, blue) hemoglobin in response to a 16s mechanical whisker stimulation in trials with no concurrent locomotion occurring between the 4 seconds either side of the stimulus period (dotted lines) for wild-type (WT, purple), APP/PS1 (AD, green), atherosclerosis (ATH, pink), and mixed APP/PS1 x atherosclerosis (MIX, orange) mice were also shown for the artery (top, **A**.), vein (middle, **D**.) and parenchyma (bottom, **G.**) regions of interest (which were taken from within the larger whisker barrel ROI). Findings were consistent with those observed in Figure 1, and showed no significant impact of disease group on the size of HbT and HbR responses (**B-C, E-F, H-I**).

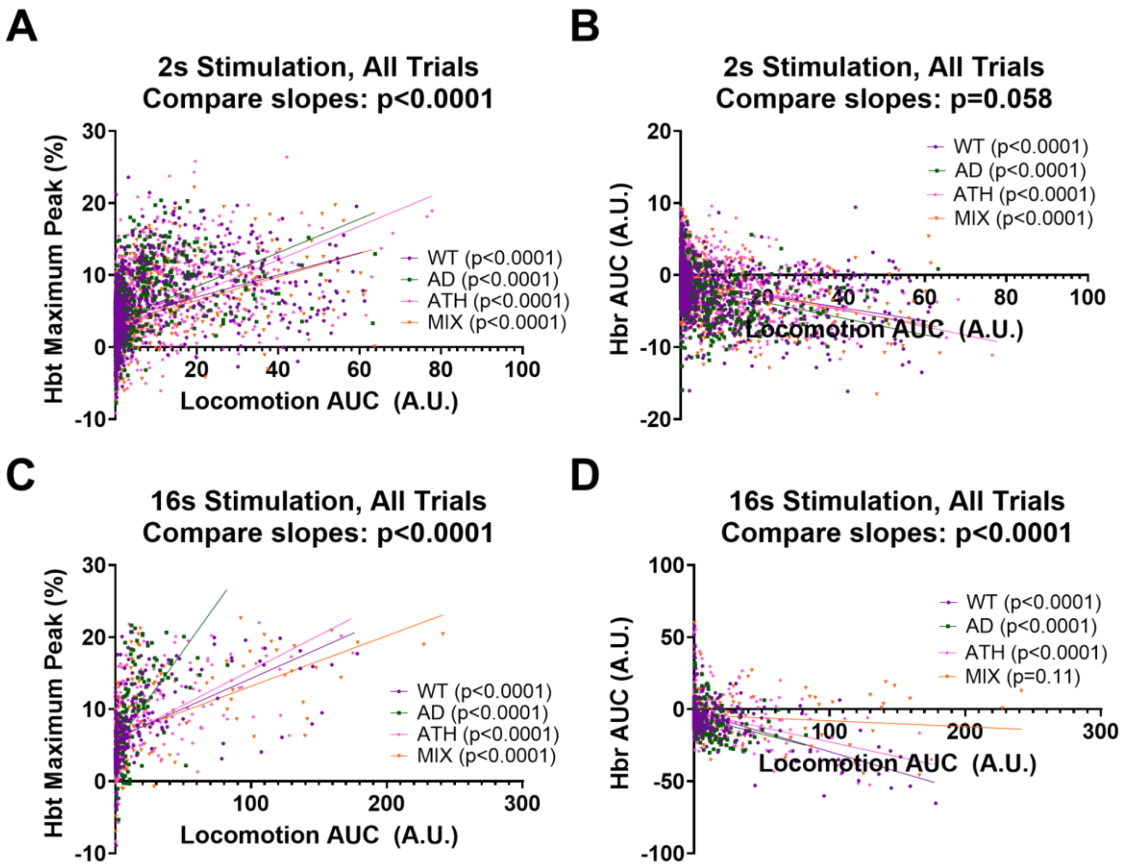

S Fig. 3 Correlating locomotion and hemodynamic response

The impact of locomotion on hemodynamic responses was visualised across all trials using scatterplots for the 2s-stimulation induced **A.** HbT maximum peak and **B.** HbR area under the curve, and the 16s-stimulation induced **C.** HbT maximum peak and **D.** HbR area under the curve. Across all scatterplots, Pearson's correlations were conducted within each disease group to assess whether locomotion and hemodynamic responses correlated (p-values displayed in legend), and simple linear regressions conducted to look for an overall effect of disease (comparing slopes between groups, p-values displayed in the title). There was a consistent effect of locomotion on hemodynamic responses across all groups, with locomotion increasing the size of responses.

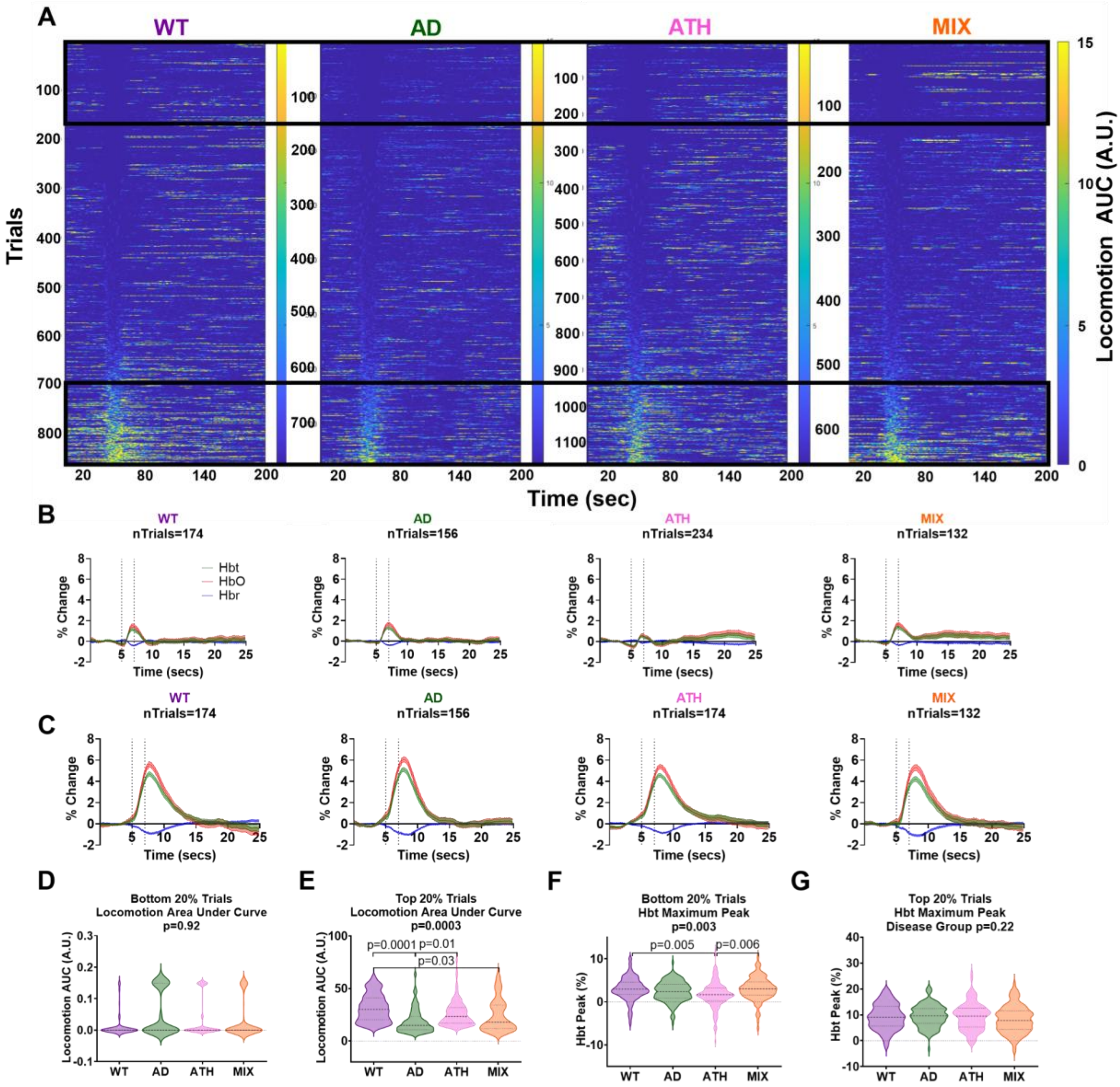

S Fig. 4 Assessing the impact of locomotion on 2s-stimulus induced hemodynamic responses across ranked trials.

An alternative method of assessing the impact of locomotion on hemodynamic responses was explored (vs Figures 2 and 4), as **A.** individual trials were ranked in ascending order from least to most locomotion during the stimulation period for WT (left, purple), AD (centre left, green), ATH (centre right, pink) and MIX (right, orange) mice. The hemodynamic responses to a 2s-whisker stimulation where then visualised for the **B.** bottom and **C.** top 20% of ranked trials across all disease groups. Across disease groups the size of locomotion responses was **D.** not different in the bottom 20% of trials (as there was no running for any of the groups), **E.** but was significantly different in the top 20% of trials (p=0.0003), where WT animals ran more than the other groups. However, using this alternative method of understanding the impact of locomotion on hemodynamic responses, findings were consistent to those observed previously, with **F.** a significant effect of disease observed in the size of the HbT response when no locomotion was present (p=0.003) due to atherosclerosis mice showing smaller responses, and **G.** no effect of disease on HbT responses impacted by locomotion (top 20% of trials).

Appendix B: Statistical Reports

Statistics Reports (SR1-SR6): The following tables report the mean, standard deviation, type of statistical test, test statistics, degrees of freedom and p-value for each statistical test reported in the main manuscript.

SR1: Figure 2: Comparing stimulation-induced responses without the confounds of locomotion. The time series metrics across disease groups (4 levels: WT, AD, ATH, MIX) for the stimulus-induced responses in the artery ROI from the whisker barrel region where compared in figures 2b-d & f-h using a linear mixed model (lmer package RStudio) with disease group as the independent variable, the dependent variable as HbT maximum peak (b,f), HbR area under the curve (c,g) or locomotion area under the curve (d,h), and animal ID as the random factor (to account for variations between groups being driven by a single outlier animal). The formula used for the linear mixed model in RStudio was: model <- lmer(DV~IV + (1|animalID), data = data_table). With DV representing the dependent variable (e.g. HbT maximum peak or HbR area under the curve for artery ROI, or locomotion area under curve) and IV the independent variable (here, genotype group). Where data (DV) was not normally distributed (test using Shapiro-Wilks test), the residuals (resid(model)) were plotted against fitted values (qqnorm(residuals)) to confirm these were normally distributed and the LMM was still robust. To further investigate where the specific significant differences were pairwise comparisons (with correction for multiple comparisons) were conducted using the Tukey method (emmeans function RStudio). The formula used for the post-hoc comparisons in RStudio was: em_res = emmeans(model, ~ IV). With the model being specified above using the lmer function, and IV again representing the independent variable.

**Figure**	**Mean**	**SD**	**Assumptions**	**Test**	**Test Statistic**	**Mean Square**	**Degrees of freedom**	**P Value**	**Post-hoc comparisons:**
2b (HbT max peak, 5-10s)<br><br>N=743 trials total	WT: 3.256, AD: 2.985, ATH: 1.905, MIX: 3.310	WT: 2.441, AD: 2.195, ATH: 2.597, MIX: 2.590	22 outlier trials, 1 extreme outlier<br><br>Shapiro-Wilks: W=0.98, p=3e-9	Linear mixed model (fixed factor: disease group)	F=4.97	28.689	3	0.0081	AD-ATH p=0.1195, AD-MIX p=0.8210, AD-WT p=0.8886, ATH-MIX p=0.0267, ATH-WT p=0.0179, MIX-WT p=0.9945
2c (HbR AUC, 5-10s)<br><br>N=743 trials total	WT: -1.261, AD: -1.275, ATH: -0.3855, MIX: -	WT: 1.899, AD: 2.015, ATH: 2.163, MIX: 1.942	20 outlier trials, 1 extreme outlier<br><br>Shapiro-Wilks: W=0.97, p=1e-10	Linear mixed model (fixed factor: disease group)	F=3.746	14.593	3	0.0303	AD-ATH p=0.0547, AD-MIX p=0.5851, AD-WT p=1.00, ATH-MIX p=0.7145, ATH-WT p=0.0416, MIX-WT p=0.5536

	0.611 3								
2d (Locomotion AUC, 5-10s) N=743 trials total	WT: 0.08118, AD: 0.08145, ATH: 0.09893, MIX: 0.06603	WT: 0.1282, AD: 0.1213, ATH: 0.1416, MIX: 0.1163	18 outlier trials, 5 extreme outliers Shapiro-Wilks: W=0.67, p=2e-16	Linear mixed model (fixed factor: disease group)	F=1.2738	0.021166	3	0.3115	AD-ATH p=0.6468, AD-MIX p=0.9403, AD-WT p=0.9999, ATH-MIX p=0.3360, ATH-WT p=0.5210, MIX-WT p=0.9465
2f (HbT max peak, 10-30s) N=146 trials total	WT: 4.563, AD: 3.540, ATH: 3.329, MIX: 4.328	WT: 3.033, AD: 1.288, ATH: 2.594, MIX: 3.768	7 outlier trials, 0 extreme outliers Shapiro-Wilks: W=0.94, p=5e-6	Linear mixed model (fixed factor: disease group)	F=0.6258	4.1957	3	0.6058	AD-ATH p=0.9967, AD-MIX p=0.9226, AD-WT p=0.8920, ATH-MIX p=0.7655, ATH-WT p=0.6445, MIX-WT p=1.00
2g (HbR AUC, 10-30s) N=146 trials total	WT: -5.667, AD: -4.17, ATH: -1.337, MIX: 1.375	WT: 6.815, AD: 5.511, ATH: 10.07, MIX: 14.94	7 outlier trials, 2 extreme outliers Shapiro-Wilks: W=0.79, p=3e-13	Linear mixed model (fixed factor: disease group)	F=3.3007	302.19	3	0.05927	AD-ATH p=0.7404, AD-MIX p=0.3326, AD-WT p=0.9535, ATH-MIX p=0.7144, ATH-WT p=0.1983, MIX-WT p=0.0632
2h (Locomotion AUC, 10-30s)	WT: 0.1447, AD: 0.2929, ATH: 0.1634, MIX:	WT: 0.1888, AD: 0.2381, ATH: 0.1790, MIX:	2 outlier trials, 0 extreme outliers Shapiro-Wilks: W=0.79	Linear mixed model (fixed factor: disease group)	F=1.9185	0.05847	3	0.1978	AD-ATH p=0.3405, AD-MIX p=0.1363, AD-WT p=0.2522, ATH-MIX p=0.7748, ATH-WT p=0.9905, MIX-WT p=0.8950

N=146 trials total	0.1032	0.1405	, p=3e-13						

SR2: Figure 3: Differences across disease models in the size of 2s-stimulation responses during rest are not linked to the high number of trials. The impact of trial number on the size of HbT responses (HbT maximum peak or HbR area under the curve) was assessed using a linear regression across disease groups. Generally for each mouse group (WT, AD, ATH, MIX) there was no relationship between the size of the HbT response and the trial number (see is the slope significantly non-zero), except for the wild-type animals in the size of the HbT maximum peak which increased as trial number increased (positive correlation), and for the MIX animals in the size of the HbR AUC which increased (larger decrease) as the trial number increased (negative correlation). There was no overall significant difference between the disease groups on the relationship between locomotion and the HbT responses (see are the slopes equal), but the intercepts were significantly different between groups (see are the intercepts equal) as the HbT maximum peak values were generally smaller for ATH mice (intercepts y-axis at lower value), and HbR AUC values generally smaller in the earlier trials for the MIX mice (intercepts y-axis closer to 0).

**Figure**	**Test**	**Is the slope significantly non-zero?**	**Are the slopes equal?**	**Are the intercepts equal?**
3a HbT maximum peak/ trial number	Linear Regression	WT: F(1,220)=5.160, p=0.0241<br><br>AD: F(1,130)=0.6367, p=0.4263<br><br>ATH: F(1,260)=0.02847, p=0.8661<br><br>MIX: F(1,125)=2.466, p=0.1188	F(3,735)=1.799, p=0.1460	F(3,738)=15.20, p<0.0001

3d HbR AUC / trial number	Linear Regression	WT: F(1,220)=2.391, p=0.1235<br><br>AD: F(1,130)=0.07866, p=0.7796<br><br>ATH: F(1,260)=0.6943, p=0.4055<br><br>MIX: F(1,125)=7.138, p=0.0086	F(3,735)=1.130, p=0.3360	F(3,738)=10.00, p<0.0001

SR3: Figure 3: Differences across disease models in the size of 2s-stimulation responses during rest are not linked to the high number of trials. The impact of disease group (WT, AD, ATH, MIX) and trial number on the size of HbT and HbR responses was assessed by categorizing data as belonging to early (trials 1-5) or late (trials 25-30) trials, and comparing the size of the responses (3b. maximum peak for HbT, and 3e. area under the curve for HbR). A linear mixed model (lmer package RStudio) was conducted with disease group and trial group (early or late) as the independent variables, and the dependent variable as HbT maximum peak (b) or HbR area under the curve (e), and animal ID as the random factor (to account for variations between groups being driven by a single outlier animal). To further investigate where the specific trend-level differences were pairwise comparisons (with correction for multiple comparisons) were conducted using the Tukey method (emmeans function RStudio). Through these linear mixed models we observed no significant impact of trial number on the size of hemodynamic responses.

Figure	Mean	SD	Test	Test Statistic	Mean Square	Degrees of freedom	P Value	Post-hoc comparisons:

3b (HbT maximum peak)	WT early: 2.022, AD early: 3.266, ATH early: 2.553, MIX early: 3.264, WT late: 3.801, AD late: 2.969, ATH late: 2.412, MIX late: 3.835	WT early: 2.733, AD early: 1.778, ATH early: 3.098, MIX early: 2.242, WT late: 2.534, AD late: 1.720, ATH late: 1.780, MIX late: 2.835	Linear mixed model (fixed factors: disease group* trial number)	Disease group: 0.9297, trial group: 1.6510, Disease group * trial group: 2.3865	Disease group: 4.9543, trial group: 8.7977, Disease group * trial group: 12.7167	Disease group: 3, trial group: 1, Disease group * trial group: 3	Disease group: p=0.4416, trial group: p=0.200, Disease group * trial group: p=0.0696	AD Early - ATH Early p=0.9928<br><br>AD Early - MIX Early p=1.0000<br><br>AD Early - WT Early p=0.8890<br><br>AD Early - AD Late p=0.9999<br><br>AD Early - ATH Late p=0.9585<br><br>AD Early - MIX p=0.9947<br><br>AD Early - WT p=0.9940<br><br>ATH Early - MIX p=0.9943<br><br>ATH Early - WT Early p=0.9966<br><br>ATH Early - AD Late p=0.9999<br><br>ATH Early - ATH Late p=0.9999<br><br>ATH Early - MIX Late p=0.6818<br><br>ATH Early - WT Late p=0.5760<br><br>MIX Early - WT Early p=0.9238<br><br>MIX Early - AD Late p=0.9999<br><br>MIX Early - ATH Late p=0.9741<br><br>MIX Early - MIX Late p=0.9988<br><br>MIX Early - WT Late p=0.9997<br><br>WT Early - AD Late p=0.9691<br><br>WT Early - ATH Late p=0.9998<br><br>WT Early - MIX Late p=0.4089<br><br>WT Early - WT Late p=0.1077

									AD Late - ATH Late p=0.9955<br><br>AD Late - MIX Late p=0.9337<br><br>AD Late - WT Late p=0.9122<br><br>ATH Late - MIX Late p=0.4816<br><br>ATH Late - WT Late p=0.3557<br><br>MIX Late - WT Late p=1.0000

3e (HbR AUC)	WT early: -1.026, AD early: -0.7663, ATH early: -0.3487, MIX early: -0.3744, WT late: -1.471, AD late: -1.144, ATH late: -0.8161, MIX late: -1.258	WT early: 2.227, AD early: 1.337, ATH early: 2.360, MIX early: 1.453, WT late: 1.895, AD late: 1.325, ATH late: 2.022, MIX late: 2.259	Linear mixed model (fixed factors: disease group* trial number)	Disease group: 1.1221, trial group: 3.4293, Disease group * trial group: 0.1470	Disease group: 4.199, trial group: 12.8354, Disease group * trial group: 0.5502	Disease group: 3, trial group: 1, Disease group * trial group: 3	Disease group: p=0.36178, trial group: p=0.06521, Disease group * trial group: p=0.93155	AD Early - ATH Early -0.3913 0.592 87.2 -0.661 0.9978<br>AD Early - MIX Early p=0.9999<br>AD Early - WT Early p=1.0000<br>AD Early - AD Late p=0.9979<br>AD Early - ATH Late p=1.0000<br>AD Early - MIX Late p=0.9939<br>AD Early - WT Late p=0.8943<br>ATH Early - MIX Early p=1.0000<br>ATH Early - WT Early p=0.9628<br>ATH Early - AD Late p=0.8357<br>ATH Early - ATH Late p=0.9942<br>ATH Early - MIX Late p=0.7284<br>ATH Early - WT Late p=0.2564<br>MIX Early - WT Early p=0.9972<br>MIX Early - AD Late p=0.9833<br>MIX Early - ATH Late p=1.0000<br>MIX Early - MIX Late p=0.9381<br>MIX Early - WT Late p=0.8132<br>WT Early - AD Late p=1.0000<br>WT Early - ATH Late p=0.9995<br>WT Early - MIX Late p=0.9998<br>WT Early - WT Late p=0.9648

								AD Late - ATH Late p=0.9877<br><br>AD Late - MIX Late p=1.0000<br><br>AD Late - WT Late p=0.9962<br><br>ATH Late - MIX Late p=0.9587<br><br>ATH Late - WT Late p=0.6138<br><br>MIX Late - WT Late p=0.9996

SR4: Figure 4: Comparing stimulation-induced responses which contain concurrent locomotion. The time series metrics across disease groups (4 levels: WT, AD, ATH, MIX) for the stimulus-induced responses in the artery ROI from the whisker barrel region during a 2s or 16s stimulation were compared in figures 4b-d & f-h using a linear mixed model (lmer package RStudio) with disease group as the independent variable, the dependent variable as HbT maximum peak (b,f), HbR area under the curve (c,g) or locomotion area under the curve (d,h), and animal ID as the random factor (to account for variations between groups being driven by a single outlier animal). The data was filtered so only trials where concurrent locomotion occurred during the stimulation period were included. The formula used for the linear mixed model in RStudio was: model <- lmer(DV~IV + (1|animalID), data = data_table filtered to include locomotion trials only). With DV representing the dependent variable (e.g. HbT maximum peak or HbR area under the curve for artery ROI, or locomotion area under curve) and IV the independent variable (here, genotype group). Where data (DV) was not normally distributed (test using Shapiro-Wilks test), the residuals (resid(model)) were plotted against fitted values (qqnorm(residuals)) to confirm these were normally distributed and the LMM was still robust. To further investigate where the specific significant differences were pairwise comparisons (with correction for multiple comparisons) were conducted using the Tukey method (emmeans function RStudio). The formula used for the post-hoc comparisons in RStudio was: em_res = emmeans(model, ~ IV). With the model being specified above using the lmer function, and IV again representing the independent variable.

**Figure**	**Mean**	**SD**	**Assumptions**	**Test**	**Test Statistic**	**Mean Square**	**Degrees of freedom**	**P Value**	**Post-hoc comparisons:**
4b (HbT max peak, 5-10s)<br><br>N=746 trials	WT: 7.917, AD: 7.257, ATH: 7.196, MIX: 7.058	WT: 5.318, AD: 4.536, ATH: 5.387, MIX: 5.006	2 outlier trials, 0 extreme outliers<br><br>Shapiro-Wilks: W=0.98, p=8e-10	Linear mixed model (fixed factor: disease group)	F=0.4526	10.685	3	0.7177	AD-ATH p=0.9984, AD-MIX p=0.9995, AD-WT p=0.7778, ATH-MIX p=0.9931, ATH-WT p=0.8236, MIX-WT p=0.7538
4c (HbR AUC, 5-10s)<br><br>N=746 trials	WT: -3.520, AD: -3.314, ATH: -2.465, MIX: -3.313	WT: 3.373, AD: 2.736, ATH: 2.947, MIX: 3.411	8 outlier trials, 0 extreme outliers<br><br>Shapiro-Wilks: W=0.99, p=2e-5	Linear mixed model (fixed factor: disease group)	F=0.8817	7.5197	3	0.4634	AD-ATH p=0.6520, AD-MIX p=0.9988, AD-WT p=0.9911, ATH-MIX p=0.7953, ATH-WT p=0.4543, MIX-WT p=0.9742

4d (Locomotion AUC, 5-10s) N=746 trials	WT: 10.55, AD: 6.574, ATH: 8.593, MIX: 9.151	WT: 13.67, AD: 8.509, ATH: 10.17, MIX: 11.57	46 outlier trials, 9 extreme outliers Shapiro-Wilks: W=0.75, p=2e-16	Linear mixed model (fixed factor: disease group)	F=1.248	145.26	3	0.3129	AD-ATH p=0.8100, AD-MIX p=0.5737, AD-WT p=0.2783, ATH-MIX p=0.9413, ATH-WT p=0.6997, MIX-WT p=0.9790
4f (HbT max peak, 10-30s) N=331 trials	WT: 9.602, AD: 8.250, ATH: 8.731, MIX: 9.128	WT: 5.815, AD: 5.357, ATH: 5.257, MIX: 6.230	0 outlier trials, 0 extreme outliers Shapiro-Wilks: W=0.98, p=5e-5	Linear mixed model (fixed factor: disease group)	F=0.1708	4.2858	3	0.915	AD-ATH p=0.9999, AD-MIX p=0.9927, AD-WT p=0.9310, ATH-MIX p=0.9941, ATH-WT p=0.9213, MIX-WT p=0.9919
4g (HbR AUC, 10-30s) N=331 trials	WT: -11.18, AD: -8.719, ATH: -7.927, MIX: -8.306	WT: 18.61, AD: 11.58, ATH: 14.80, MIX: 16.04	29 outlier trials, 4 extreme outliers Shapiro-Wilks: W=0.95, p=10e-9	Linear mixed model (fixed factor: disease group)	F=0.3598	74.169	3	0.7825	AD-ATH p=0.9936, AD-MIX p=0.9988, AD-WT p=0.9247, ATH-MIX p=0.9998, ATH-WT p=0.7551, MIX-WT p=0.8796
2h (Locomotion AUC, 10-30s) N=331 trials	WT: 24.02, AD: 9.038, ATH: 17.88, MIX: 32.93	WT: 38.65, AD: 10.79, ATH: 28.51, MIX: 59.68	38 outlier trials, 0 extreme outliers Shapiro-Wilks: W=0.58, p=2e-16	Linear mixed model (fixed factor: disease group)	F=0.9565	840.14	3	0.4295	AD-ATH p=0.8595, AD-MIX p=0.3891, AD-WT p=0.6554, ATH-MIX p=0.7209, ATH-WT p=0.9606, MIX-WT p=0.9376

SR5: Figure 5: No differences in performance on a novel object recognition task between disease groups. For the distance run during training and testing (5b), velocity during training and testing (5c), and the preference index (5d) (dependent variables) the data was compared across disease groups (independent variable, 4 levels: WT, AD, ATH, MIX). All datasets were first tested for normality using the Shapiro-Wilks test (e.g. Figure 5b distance run the following individual groups were each tested: training WT, testing WT, training AD, testing AD, training ATH, testing ATH, training MIX, testing MIX), and for variance between groups using the Brown-Forsythe test (distance

run training p=0.98, distance run testing p=0.92, velocity training p=0.75, velocity testing p=0.88, preference index p=0.95), with non-significant values indicating the assumptions of the one-way ANOVAs had been met. When the Shapiro-Wilks was run across the entirety of the dataset (i.e. collapsed across groups), for the distance run (cm) testing dataset, the data was not normally distributed and so a Kruskal-Wallis test was run as the non-parametric equivalent to the one-way ANOVA. We saw no significant differences in performance on the novel object recognition task between disease groups. Due to the AD mice performing below chance for the preference index (5d) we have also displayed the Tukey post-hoc comparisons to highlight we still detect no differences between the AD mice or any other mouse groups.

**Figure**	**Mean**	**SD**	**Assumptions**	**Test**	**Test Statistic**	**Degrees of freedom**	**P Value**
5b (distance run, cm)	Training:<br><br>WT: 2437, AD: 2482, ATH: 2815, MIX: 2828<br><br>Testing:<br><br>WT: 1982, AD: 2057, ATH: 2239, MIX: 2083	Training:<br>WT: 581.5, AD: 390.6, ATH: 531.6, MIX: 546.6<br><br>Testing:<br><br>WT: 492.2, AD: 484, ATH: 752.9, MIX: 761.7	Training: 1 outlier, 0 extreme. Shapiro-Wilks: W=0.96, p=0.38.<br><br>Testing: 2 outlier, 0 extreme. Shapiro-Wilks: W=0.90, p=0.01.	One-way ANOVA for training<br><br>Kruskal-Wallis for testing	Training: F=1.107<br><br>Testing:<br><br>Chi-squared= 0.45	DFn=3, DFd=24	Training: p=0.3657<br><br>Testing: p=0.9303
5c (velocity, cm/s)	Training:<br><br>WT: 4.358, AD: 4.612, ATH: 5.098, MIX: 4.85<br><br>Testing:<br><br>WT: 3.539, AD: 3.571, ATH: 4.00, MIX: 3.752	Training:<br>WT: 1.083, AD: 1.353, ATH: 1.137, MIX: 0.9114<br><br>Testing:<br><br>WT: 1.067, AD: 0.996, ATH: 1.333, MIX: 1.487	Training: 1 outlier, 0 extreme. Shapiro-Wilks: W=0.93, p=0.06.<br><br>Testing: 2 outlier, 0 extreme. Shapiro-Wilks: W=0.93, p=0.06.	One-way ANOVA	Training: F=0.5918<br><br>Testing:<br><br>F=0.2293	DFn=3, DFd=24	Training: p=0.6264<br><br>Testing: p=0.8751
5d (preference index)	WT: 58.2, AD: 48.94, ATH: 61.46, MIX: 56.95	WT: 17.06, AD: 13.49, ATH: 13.12, MIX: 17.42	0 outliers, 0 extreme outliers<br><br>Shapiro-Wilk test	One-way ANOVA	F=0.8800	DFn=3, DFd=24	p=0.4653

nAnimals= 28			(across all variables) : W=0.95, p=0.21				
	Tukey posthoc comparison: WT-AD p=0.7129; WT -ATH p=0.9744; WT-MIX p=0.9989; AD-ATH p=0.3909; AD-MIX p=0.7934; ATH-MIX p=0.9367						

SR6: Figure 6: No differences in pathology across disease models. To assess pathology between our disease models we compared amyloid plaque coverage (% area) in AD and MIX mice, and aortic arch plaque load in ATH and MIX mice. The Shapiro-Wilks test of normality and Brown-Forsythe or F test of independence were conducted for each group to check the t-test or ANOVA assumptions were met, and all contained data which was normally distributed (6b: ATH: p=0.74, MIX: p=0.45; 6d: AD cortex p=0.73, MIX cortex p=0.62, AD hippocampus p=0.87, MIX hippocampus p=0.10) and samples independent (6b p=0.25; 6d p=0.40). As the aortic arch comparison had only two levels (independent variable disease group, 2 levels: ATH, MIX) an unpaired t-test was conducted; whereas for the amyloid beta assessment a mixed experimental design was used (disease 2 levels: AD, MIX; brain region 2 levels: cortex, HC) meaning a mixed ANOVA was conducted.

**Figure**	**Mean**	**SD**	**Test**	**Test Statistic**	**Degrees of freedom**	**P Value**
6b (aortic plaque load)	ATH: 18.26<br><br>MIX: 17.75	ATH: 4.788<br><br>MIX: 2.287	Unpaired t-test	t=0.1977	DFn=7, DFd=3	p=0.2519
6d (Abeta plaque load)	AD Cortex: 2.118<br><br>MIX Cortex: 1.489<br><br>AD HC: 2.271<br><br>MIX HC: 1.332	AD Cortex: 0.8311<br><br>MIX Cortex: 0.7746<br><br>AD HC: 0.3132<br><br>MIX HC: 0.5416	Mixed ANOVA	Genotype F=3.3470, Brain region F=0.00013, genotype * brain region F=0.7450	DFn 1, DFd 7	Genotype p=0.110, Brain region p=0.991, genotype * brain region p=0.417
